# Proteomic analysis of *Lactobacillus casei* GCRL163 cell-free extracts reveals a SecB homolog and other biomarkers of prolonged heat stress

**DOI:** 10.1371/journal.pone.0206317

**Published:** 2018-10-25

**Authors:** Kayode T. Adu, Richard Wilson, David S. Nichols, Anthony L. Baker, John P. Bowman, Margaret L. Britz

**Affiliations:** 1 Tasmanian Institute of Agriculture, University of Tasmania, Hobart, Tasmania, Australia; 2 Central Science Laboratory, University of Tasmania, Hobart, Tasmania, Australia; University of British Columbia, CANADA

## Abstract

Prolonged heat stress is one of the harsh conditions *Lactobacillus casei* strains encounter as non-starter lactic acid bacteria in dairy product manufacture. To understand the physiological and molecular mechanisms through which *Lb*. *casei* GCRL163 adapts to persistent elevated temperature, label-free quantitative proteomics of cell-free extracts was used to characterize the global responses of the strain cultured anaerobically in bioreactors at 30 to 45°C, pH 6.5, together with GC-MS for fatty acid methyl ester analysis at different growth phases. At higher growth temperatures, repression of energy-consuming metabolic pathways, such as fatty acid, nucleotide and amino acid biosynthesis, was observed, while PTS- and ABC-type transporter systems associated with uptake of nitrogen and carbon sources were up-regulated. Alkaline shock protein Asp23_2 was only detected at 45°C, expressed at high abundance, and presumptive α-L-fucosidase only at 40 and 45°C, with highly increased abundance (log_2_-fold change of 7) at 45°C. We identified a novel SecB homolog as a protein export chaperone putatively involved in posttranslational translocation systems, which was down-regulated as growth temperature increased and where the modelled 3D-structure shared architectural similarities with the *Escherichia coli* SecB protein. Membrane lipid analyses revealed temporal changes in fatty acid composition, cyclization of oleic acid to cyclopropane and novel cyclopentenyl moieties, and reduced synthesis of vaccenic acid, at higher temperatures. An 18kDa α-crystallin domain, Hsp20 family heat shock protein was more highly up-regulated in response to heat stress compared to other molecular chaperones, suggesting this protein could be a useful biomarker of prolonged heat stress in *Lb*. *casei* GCRL163.

## Introduction

*Lactobacillus casei* and related species occur in fermented milk and cheese products as non-starter, adventitious microbiota where they improve ripening and flavour development [1]. Benefits to human health, demonstrated in animal models [2, 3], have been attributed to ingestion of appropriate probiotic formulations containing *Lb*. *casei*. However, during probiotic formulation, manufacture of fermented food and passage through the gastrointestinal tract, bacteria are exposed to several potential stressors, such as high or low temperature, starvation, low pH, bile salts, and changing redox and osmotic conditions [4–6]. Heat stress is one of the most characterized and commonly encountered conditions experienced by various lactic acid bacteria (LAB) [7]. For instance, LAB in raw milk are exposed to high temperatures during pasteurization and starter cultures added for the production of different types of cheese are also subjected to heating (cooking and scalding) after pasteurization [6]. To confer probiotic and other functional traits, LAB must survive exposure to heat and other stressors during manufacturing processes, as well as harsh conditions encountered in the gastrointestinal tract following consumption. Under these conditions, several physiological and molecular responses are induced to enhance growth, adaptation and survival so alleviating impacts of stress [6].

Heat stress responses have often been studied as a rapid transient upshift in temperature whereby bacterial populations are exposed briefly to heat shock, eliciting the heat shock response which is commonly used to describe adaptive responses of bacteria to heat stress [8–10]. The heat shock response is typified by the upregulation of chaperone proteins involved in protein folding and turnover [11, 12]. Chaperone proteins are also induced by other stressors and prior heat shock pre-conditions cells to protection against subsequent challenge by other stressors [13, 14]. In practice, however, LAB populations are typically exposed to prolonged periods of heating during fermented food processing. For instance, pasteurization of raw milk involves exposing the microbiota, including LAB, to high temperatures (>60°C) for up to 30 min [6], resulting in lowered bacterial load. The cellular and physiological responses of *Escherichia coli* and *Bacillus thuringiensis* YBT-1550 to such prolonged heat stress have been reported [15, 16]. Studies of heat stress at a transcriptional level involving *Bifidobacterium breve* UCC 2003 for 150 min has also been reported [17]. However, there is a paucity of detailed information available at the transcriptomic or proteomic level on how LAB cells, in particular *Lb*. *casei*, respond to prolonged period of stress, including heat stress, and whether the underlying mechanisms differ from ‘shock’ responses. Indeed, there is growing research interest in documenting the stress physiology of LAB species under long-term exposure to stressors in context of improving the fitness of strains for fermentation and probiotic applications [6, 18]. Improvements in proteomic technologies, moving from two-dimensional-gel/mass spectrometry (MS) to high-throughput, gel-free, MS/MS systems, has greatly improved proteomic analyses, particularly for determining modulation of proteins expressed in low abundance, expanding the current knowledge of stress adaptation in *Lactobacillus* [13].

In the current investigation, we identified changes in the cellular proteome of *Lb*. *casei* GCRL163 cultured under anaerobic, pH-controlled conditions in bioreactor systems for culture temperatures of 30°C, 35°C, 40°C and 45°C. Label-free quantitative proteomic analysis of cell-free extracts (CFEs) from mid-exponential cultures and statistical analysis revealed that an increasing number of proteins were affected as the growth temperature was increased. More than 50% of the identified proteins were significantly modulated during prolonged heat stress experienced during culture at a temperature close to the maximum permissible growth temperature of 45°C, suggesting that this strain depended on the mobilization of heat stress machinery vital for protecting cellular and metabolic functions to initiate and sustain growth. Moreover, metabolic pathways appeared to be rerouted during extreme thermal stress to conserve energy, with repression of energy-consuming activities, such as fatty acid and nucleotide biosynthesis, and upregulation of enzymes in the glycolytic pathway at 45°C. In contrast, cells cultured at 40°C induced responses for maintaining cellular survival, including upregulation of most proteins in fatty acid synthesis and non-differential modulation of most proteins involved in glycolysis. Changes in fatty acid composition were observed to be growth phase- and temperature-dependent as previously reported in other bacterial species [19, 20], with reduced vaccenic acid synthesis in cells from 45°C culture and detection of cyclopropane derivatives of C18:0 fatty acids and previously unreported cyclopentenyl moieties.

## Materials and methods

### Bacterial strain and growth conditions

The bacterial strain, *Lb*. *casei* GCRL163, originally isolated from Cheddar cheese during maturation, was stored at -80°C in De Man, Rogosa and Sharpe (MRS) broth (Oxoid, Australia) containing 40% glycerol [21]. The strain was plated onto MRS agar and incubated anaerobically using an AnaerocultR A system (Sigma-Aldrich, Australia) at 37°C for 48 h. Starter cultures were routinely prepared by inoculating a single colony of the strain into freshly prepared MRS broth prior to anaerobic incubation at 37°C for 12 h.

### Prolonged heat stress in the bioreactor systems

A set of four water-jacketed Benchtop bioreactor systems (New Brunswick, Eppendorf, USA) each with 800 mL of MRS broth were supplemented with an additional 1% glucose. The temperatures were maintained at 30°C, 35°C, 40°C and 45°C and monitored by a resistance temperature detector (RTD) submerged in a thermowell containing glycerol, with set points maintained through the automated control systems. The media were inoculated to an optical density at 600 nm (OD_600_) of 0.02 from overnight starter cultures (MRS broth, 12 h, 37°C). The bioreactors were maintained at an agitation of 50 rpm. Anaerobic conditions were maintained by sparging with nitrogen gas at 100% set-point using ring sparger at 0.5 SLPM gas flow while oxygen, carbon dioxide and air were maintained at 0% set-point before inoculation and during growth. Oxygen level was monitored using dissolved oxygen (dO_2_) probes and the pH set point maintained at 6.5 with automatic online addition of 2M NaOH. All set-points were programmed from BioFlo/CelliGen 115 control stations and the loops monitored using BioCommand with OPC computer software (Eppendorf, Australia).

### Cell lysis and protein extraction

Cultures were harvested at mid-exponential growth phase [7 h (30°C), 6 h (35°C), 5 h (40°C) and 4 h (45°C)] corresponding to OD_600_ 1.04, 1.50, 0.69 and 0.19 respectively (S1 Fig) by centrifuging at 9,000 rcf (Eppendorf centrifuge 5810R) for 10 min at 20°C and used for CFE preparation following the methods described by Nezhad *et al*. [22]. The cells were washed once with Tris-HCl buffer (0.04 M, pH 7.0) and then resuspended in an appropriate volume of Tris-HCl buffer (0.04 M, pH 7.0) to obtain an OD_600_ of ~ 20 and stored at -20°C. Triplicate samples were taken from each bioreactor unit at different temperatures for the proteomic analysis. CFEs were prepared by mixing 0.5 mL of the cell suspension with 1 g Zircon beads (0.1 mm diameter; Daintree Scientific Pty Ltd, Australia) in 2 mL screw-capped plastic tubes and then subjected to ten bursts of 1 min followed by 1 min cooling on ice between bursts using a TissueLyser II (Qiagen, Australia). Cell debris was removed by centrifugation at 14,000 rcf for 30 min at 4°C (Eppendorf centrifuge 5417R, Crown Scientific Pty Ltd, Australia) and supernatant fluid collected and stored at -80°C until analysis. Protein concentrations were determined using the Bradford protein assay [23].

### Lipid extraction and fatty acid analysis

This was as previously described and applied with slight modifications [24]. Briefly, 50 mg wet weight of the cells collected at mid-log, late log and stationary phases were washed twice with Tris-HCl buffer (0.04 M, pH 7.0) to remove residual MRS medium. Cells were resuspended in 3 mL of esterification reagent (10:1:1 [v/v] methanol:chloroform:HCl [Sigma-Aldrich, Australia]) followed by the addition of 100 μL of surrogate working standard (500 μg/mL nonadecanoic acid in chloroform [Sigma-Aldrich, Australia]), then the mixture was heated at 80°C for one hour. After cooling to room temperature, 1 mL of distilled water was added then the fatty acid methyl esters (FAMEs) extracted using 1 mL of extraction solution (4:1 [v/v] hexane:chloroform [Sigma-Aldrich, Australia]). The emulsion was separated by centrifugation (Eppendorf centrifuge 5417R, Crown Scientific Pty Ltd, Australia) at 2,000 rcf for 5 min and an aliquot (2 mL) of the upper phase was then collected and dried by evaporation under nitrogen at room temperature. The dried extract was dissolved in 1 mL of internal working standard (50 μg/mL methyl tricosanoate in chloroform [Sigma-Aldrich, Australia]). Only glassware was used throughout the procedures and the cells not treated with surrogate working standard were used as control. Fatty acid compositions were then resolved and identified using gas chromatography/mass spectrometry.

### Protein identification by nanoLC-MS/MS

Protein extracts (100 μg) were precipitated with 100% ethanol (9:1 [v/v]), resuspended in 100 μL denaturation buffer (7M urea, 2M thiourea and 30mM Tris pH 8.0) and then sequentially reduced, alkylated and in-solution digested with trypsin at a protein:trypsin of 50:1 using published methods [25]. Tryptic peptides equivalent to 1 μg of each protein digest were analyzed by nanoLC-MS/MS analysis using an LTQ-Orbitrap XL and Ultimate 3,000 RSLCnano system (ThermoFisher Scientific, USA). Peptides were loaded for 3 min onto a 20 mm x 75 μm trapping column packed with PepMap100 3 μm C_18_ resin at flow rate of 0.05 mL/min using a mobile phase composed of 98% water, 2% acetonitrile and 0.05% trifluoroacetic acid. Peptides were then separated at a flow rate of 0.3 μL/min on a 250 mm 75 μm analytical column packed with PepMap 100 2μm C_18_ resin (ThermoFisher Scientific, USA) held at 40ºC. A multi-segment gradient from 98% mobile phase A (0.1% formic acid in water) to 50% mobile phase B (0.08% formic acid in 80% acetonitrile and 20% water) using three segments (3–10% B over 12 min; 10–40% B over 120 min; 40–50% B over 10 min), holding at 95% B for 20 min then re-equilibration in 3% B for 15 min was used. The LTQ-Orbitrap XL was controlled using Xcalibur 2.1 (ThermoFisher Scientific, USA) and operated in data-dependent acquisition mode where survey scans (m/z 460–2,000) were acquired in the Orbitrap at a resolving power of 60,000. MS/MS spectra were concurrently acquired in the LTQ mass analyzer on the eight most intense ions from the FT survey scan. Unassigned and singly-charged precursor ions were not selected for fragmentation and 30-sec dynamic exclusion (repeat count 1 exclusion list size 500) was used. Fragmentation conditions in the LTQ were: 35% normalized collision energy, activation q of 0.25, 30 min activation time and minimum ion selection intensity of 3,000 counts.

### Database searching and statistical analysis of identified proteins

RAW files from the LTQ-Orbitrap were imported into MaxQuant software version 1.5.1.2 (http://www.maxquant.org/) using the extracted ion currents of matched peptides [26]. The extracted MS/MS spectra were searched against the UniProt proteome for *Lb*. *casei* strain W56 (ID UP000003734) database comprising 3,092 protein entries (www.uniprot.org) using the Andromeda search engine, since the genome of *Lb*. *casei* GCRL163 has been shotgun sequenced (DDBJ/EMBL/GenBank accession number MODT00000000) but not closed [27]. Hence, *Lb*. *casei* W56 was used as the reference for *Lb*. *casei* GCRL163 proteins as the two strains are phylogenetically closely clustered on the basis of pfam analysis and the genome of strain W56 is closed. The MaxQuant default parameters for protein identification by LTQ-Orbitrap MS/MS and label-free quantitation (LFQ) were used, including a maximum of two missed trypsin cleavages, variable oxidation of methionine and fixed carbamidomethylation of cysteine. The false discovery rates for peptide-spectrum matches and protein identification were both set to 1%. MaxLFQ was used for peptide intensity determination and normalization, based on pair-wise comparison of unique and razor peptide intensities and a minimum ratio count of 2 [28]. The *ProteinGroups*.*txt* output file generated by MaxQuant was processed as follows: the normalised label-free quantification (LFQ) peptide intensity values, the numbers of razor and unique peptides for each of the identified proteins were imported into Perseus software version 1.5.031 (http://perseus-framework.org/). Protein groups identified as potential contaminants (prefixed with CON_) and proteins identified only by site or by reverse database matching were removed and remaining observed intensity values were log_2_ transformed. Proteins were filtered to exclude proteins detected in fewer than nine of the biological samples or on the basis of a single matching peptide. Missing values were replaced with random intensity values for low-abundance proteins based on a normal distribution of protein abundances. To determine proteins that were significantly altered in abundance by heat treatments, a two-sided *t*-test using Benjamini Hochberg correction for multiple hypothesis testing was applied. A 1% FDR threshold was applied to determine statistical significance, unless indicated otherwise. A second dataset was generated by filtering to include proteins that were detected in a minimum of three replicates of any biological sample, to detect proteins only present at moderately high abundance at one or two specific growth temperatures. Identification of homologs to uncharacterized proteins was done through UniProt [29] and putative identity assigned where appropriate from protein sequence analyses by BLASTN through Kyoto Encyclopedia of Genes and Genomes (KEGG) [30] and the National Centre for Biotechnology Information (NCBI) [31].

### Bioinformatic analysis of identified proteins and prediction of promoters in intergenic regions

Functional profiling of the identified proteins was done according to their GO category for molecular functions based on the functional annotations of *Lb*. *casei* W56 [32]. The *t*-value for each functional group was determined by T-profiler analysis as described previously [33–35]. The *t*-values were then imported into online bioinformatics resource, Gene Cluster (version 3.0), to determine functional annotation clustering using unsupervised hierarchical cluster analysis with complete linkage. Heat maps of the T-profiler data (*t*-values) and Z-scored LFQ protein expression data were generated using Java Treeview 1.1.6R4 and Genesis software, respectively. The mass spectrometry proteomics data have been deposited to the ProteomeXchange Consortium via the PRIDE [36] partner repository with the dataset identifier PXD007097.

The BPROM tool in the Softberry program suite (www.softberry.com/) [37] was used to predict sigma-70 promoter sites in intergenic regions of the *Lb*. *casei* GCRL163 genome.

### 3D-structural modelling of SecB proteins and phylogenetic analysis

Proteins annotated as the bacterial export chaperone SecB (IPR003708) or containing a domain related to the SecB-like superfamily (IPR035958) were identified in UniProt in *E*. *coli*, *Bacillus* and *Lactobacillus* species, including reviewed and uncharacterised proteins. FASTA sequences, and the sequence of an uncharacterised protein in the genome of *Lb*. *casei* GCRL163 with IPR035958 and DUF1149 domains, were submitted to Phyre2 [38] for 3D-structural modelling, using intensive mode. Models were visualised and reviewed in Phyre2 using JSmol for secondary structures and confidence (normally >86% of residues modelled with >90% confidence) and in the UCSF Chimera package [39], with structural alignment made using the Matchmaker tool in the latter. The protein sequence of the SecB homolog of *Lb*. *casei* GCRL163 was submitted to BLASTP through KEGG to detect similar proteins in *Lactobacillus* species. FASTA sequences of these proteins, and others identified in UniProt as SecB homologs in *E*. *coli*, *Lactobacillus* and *Bacillus* species, were submitted for multi- (Clustal Omega) and pair-wise (Needle) sequence alignment (EMBL-EBI, www.ebi.ac.uk). Phylogenetic trees for identifying clades (EMBL-EBI, www.ebi.ac.uk) and hierarchical clustering (www.Phylogeny.fr) [40] were constructed.

## Results

### Quantitative and bioinformatic-functional analysis of differentially expressed proteins during prolonged heat stress

**Fig 1:**
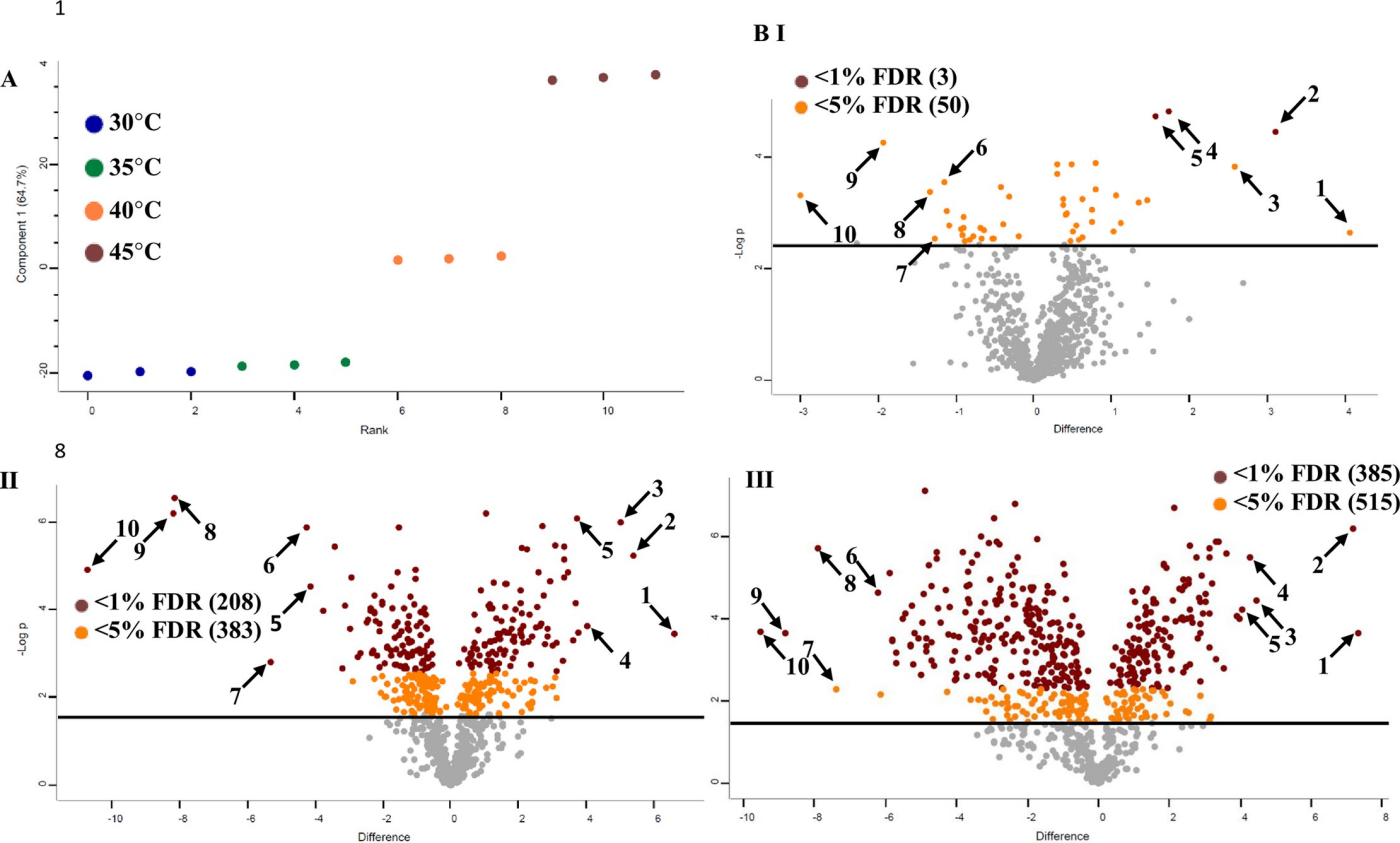
Dot plot representation of the impact of prolonged heat exposure on the proteome of *Lb*. *casei* GCRL163. (A) Principal Component Analysis (B) two-sided *t*-test analysis of the protein LFQ expression data for the temperature comparisons of (I) 35°C *vs* 30°C, (II) 40°C *vs* 30°C and (III) 45°C *vs* 30°C. The numbers of significant differentially-expressed proteins at 1% and 5% FDR thresholds after correction for multiple hypothesis testing (brown dots and orange dots, respectively) are shown in parentheses. Significant proteins with the highest fold-difference between 30°C LFQ values and 35°C, 40°C and 45°C LFQ values, respectively, are indicated by arrows and the corresponding protein identities and log_2_ fold-changes are shown in Table 1.

According to growth curves obtained for temperatures between 25°C and 55°C, the highest growth rate for strain GCRL163 was at 37°C and no significant growth occurred at temperatures above 50°C (S1 Fig). When cultured in bioreactors at 45°C, the μ_max_ was greatly reduced relative to lower temperatures and cells entered stationary phase earlier, with a lower final biomass (judged from OD_600_ readings), indicating considerable metabolic impairment of cells growing close to the maximum permissible temperature for this strain, 45°C (S1 Fig). To investigate the response of *Lb*. *casei* GCRL163 to prolonged exposure to growth-permissive but inhibitory temperatures at the proteome level, we used the MaxQuant platform for label-free quantitative (LFQ) proteomic analysis (see Materials and Methods). The peptide- and protein-level MaxQuant output files are reported in S1 Table. A high-confidence dataset, comprised of proteins detected in a minimum of 9 of the 12 biological samples and excluding proteins with <2 matching peptides, suspected contaminants and reverse database matches, was used for statistical comparison of the treatment groups. Of the 1,081 total proteins identified in the cell-free extracts, 773 proteins met these filtering criteria. Principal component analysis indicated PC1 explained 65% of the variance in the data, with increasing separation of the higher temperature samples from the 30°C samples (Fig 1A). Consistent with this, two-sample *t*-tests indicated that an increasing number of proteins were significantly impacted at increasing temperatures. For example, at an FDR threshold of 1%, 3 proteins, 285 proteins and 515 proteins were significantly affected at 35°C, 40°C and 45°C, respectively, relative to growth at 30°C (Fig 1B [I-III]). This result suggests that growth temperatures of 40°C and 45°C imposed significant thermal stress on *Lb*. *casei* GCRL163.

The results of statistical analysis of the protein LFQ expression data are shown in full in S2 Table, and the most highly differentially expressed proteins, according to the fold-differences in mean protein LFQ values at 35°C, 40°C and 45°C, respectively, relative to 30°C, are labelled numerically on the *t*-test volcano plots and summarized in Table 1.

**Table 1:** Most highly differentially altered proteins in *Lb*. *casei* GCRL163, following culture at different temperatures as annotated on the Volcano plots in Fig 1B (I-III).

**No.**	**Protein name**	**Protein ID**	**Peptides**	**MS/MS**	***p*_35/30°C**	**FDR**	**Log2_35/30°C**	Protein name*
1	**ThiM**	K0N1Q6	6	124	0.0022575	<5%	4.06	Hydroxyethylthiazole kinase
2	**BN194_29440**	K0MYM2	9	70	3.50E-05	<1%	3.10	Uncharacterized protein /Acid shock protein, alpha-crystallin, Hsp20 domain (IPR002068)*
3	**BN194_05550**	K0N257	2	11	0.0001492	<5%	2.58	Uncharacterized protein
4	**DnaJ**	K0N5J4	13	83	1.54E-05	<1%	1.74	Chaperone protein DnaJ
5	**MetE**	K0N658	24	121	1.87E-05	<1%	1.56	5-methyltetrahydropteroyl-triglutamatehomocysteine methyltransferase
6	**GlnA_2**	K0MW64	28	474	0.0002813	<5%	-1.15	Glutamine synthetase
7	**PepC**	K0NC50	14	37	0.0028795	<5%	-1.27	PepC protein
8	**Sph**	K0N5Q0	9	32	0.000424	<5%	-1.33	Sph protein
9	**Cap4C**	K0N7E3	22	220	5.47E-05	<5%	-1.94	UTP—glucose-1-phosphate uridylyltransferase
10	**PepT**	K0N213	11	33	0.0004884	<5%	-3.00	Peptidase T
**No.**	**Protein name**	**Protein ID**	**Peptides**	**MS/MS**	***p*_40/30°C**	**FDR**	**Log2_40/30°C**	**Protein name**
1	**ThiM**	K0N1Q6	6	124	0.0003666	<1%	6.61	Hydroxyethylthiazole kinase
**2**	**BN194_29440**	K0MYM2	9	70	6.01E-06	<1%	5.39	Uncharacterized protein /Acid shock protein, alpha crystallin, Hsp20 domain (IPR002068)*
3	**DppE_3**	K0NAD1	13	33	1.02E-06	<1%	5.03	DppE_3 protein
4	**BN194_19800**	K0N670	4	12	0.0002315	<1%	4.05	Uncharacterized protein /ABC transporter substrate-binding protein (IPR007487)*
5	**MetQ_2**	K0MV23	8	48	0.0003446	<1%	3.78	Lipoprotein
6	**PepC**	K0NC50	14	37	1.34E-06	<1%	-4.24	PepC protein
7	**PurK_2**	K0NB96	9	78	0.0001214	<1%	-5.31	N5-carboxyaminoimidazole ribonucleotide synthase
8	**PurD**	K0MWC4	16	177	0.0002099	<1%	-8.14	Phosphoribosylamine—glycine ligase
9	**PurH**	K0N634	18	286	1.89E-06	<1%	-8.18	Bifunctional purine biosynthesis protein PurH
10	**PurC**	K0N5S8	12	145	0.0002252	<1%	-10.73	Phosphoribosylaminoimidazole- succinocarboxamide synthase
**No.**	**Protein name**	**Protein ID**	**Peptides**	**MS/MS**	***p*_45/30°C**	**FDR**	**Log2_45/30°C**	**Protein name**
1	**ThiM**	K0N1Q6	6	124	0.0002278	<1%	7.33	Hydroxyethylthiazole kinase
2	**BN194_29440**	K0MYM2	9	70	6.34E-07	<1%	7.17	Uncharacterized protein /Acid shock protein, alpha crystallin, Hsp20 domain (IPR002068)*
3	**BN194_30140**	K0MYT6	5	14	3.49E-05	<1%	4.44	Uncharacterized protein /Aldolase-type TIM barrel (IPR013785)
4	**FruA_3**	K0N4S1	10	78	3.23E-06	<1%	4.26	FruA_3 protein
5	**BN194_30000**	K0N979	7	21	5.77E-05	<1%	4.07	Uncharacterized protein /Glycosyl transferase family 1 (IPR001296)*
6	**PepC**	K0NC50	14	37	2.26E-05	<1%	-6.19	PepC protein
7	**OppA_2**	K0MWL6	19	174	0.0051703	<5%	-7.39	OppA_2 protein
8	**PurH**	K0N634	18	286	1.89E-06	<1%	-7.90	Bifunctional purine biosynthesis protein PurH
9	**PurC**	K0N5S8	12	145	0.0002252	<1%	-8.83	Phosphoribosylaminoimidazole-succinocarboxamide synthase
10	**PurD**	K0MWC4	16	177	0.0002099	<1%	-9.53	Phosphoribosylamine—glycine ligase

*UniProt submitted or reviewed name for *Lb*. *casei* W56 proteins; domains which indicate potential function are specified in parentheses.

**Fig 2:**
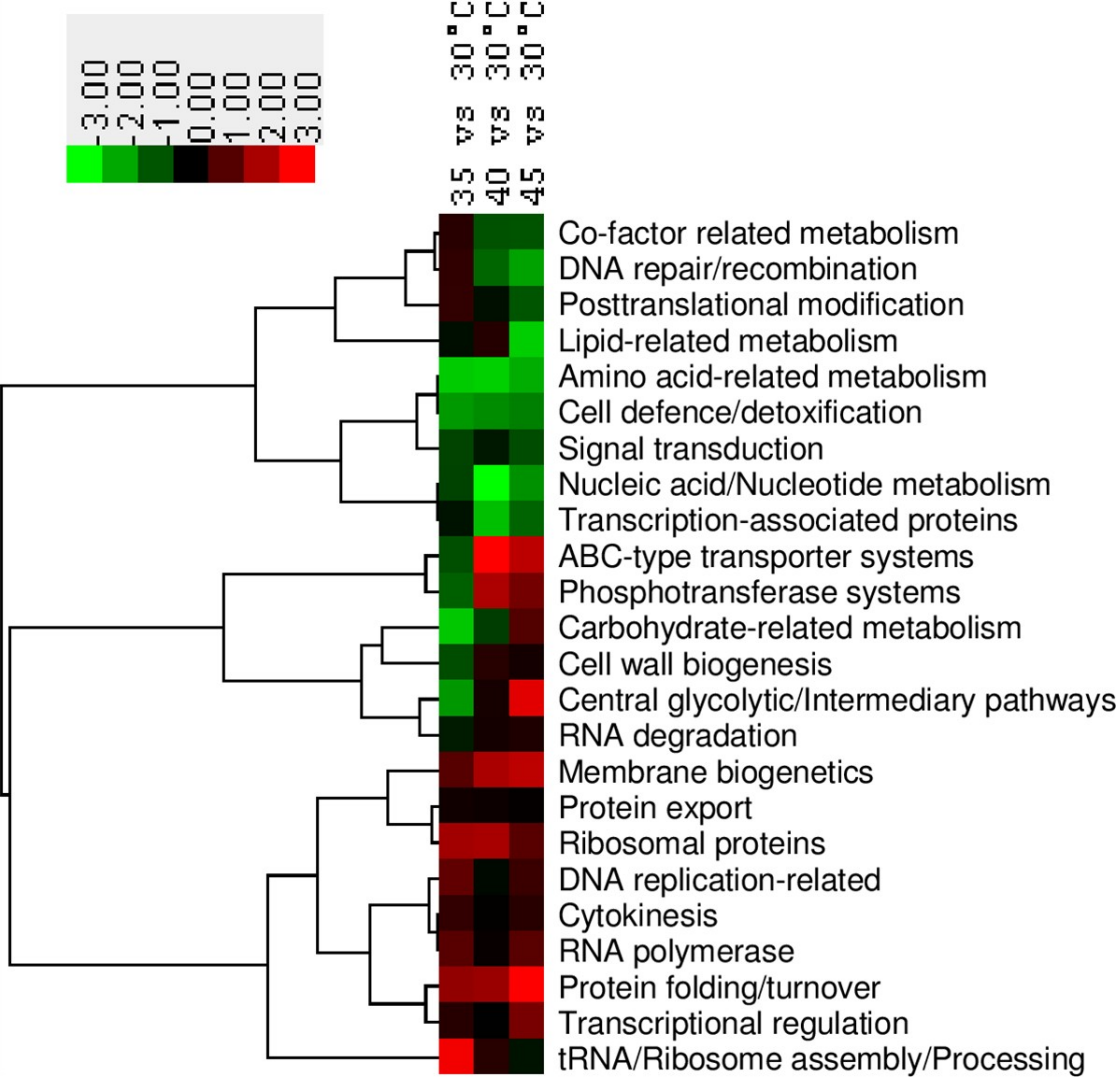
Heat map depicting change in abundance trends in functional groups of proteins in *Lb*. *casei* GCRL163 based on *t*-test values derived from T-profiler analysis at different temperatures (35°C, 40°C, 45°C compared to 30°C) using cluster v.3.0 software. The overall proteomic data at different temperatures (top) were compared to the protein functional groups (left dendogram) using unsupervised hierarchical clustering on the basis of uncentered correlation. Analysis of the proteins based on the functional classification as indicated by the colour scale based on *t*-values revealed differentially expressed proteins.

To gain a global overview of the protein functional classes impacted by thermal stress, proteins in the filtered set of 773 were grouped according to GO categories for molecular function based on the functional annotations of strain *Lb*. *casei* W56 and changes in abundance relative to 30°C were analysed using T-profiler (S3 Table). Clustering the data according to *t*-values revealed protein functional classes with consistent trends across all three elevated temperatures (either consistently up- or down-regulated) and those with more complex patterns of regulation (Fig 2). For example, proteins associated with the terms membrane bioenergetics (*n* = 12) and protein folding and turnover (*n* = 22) were consistently more abundant at growth temperatures of 35°C, 40°C and 45°C relative to 30°C. In contrast, proteins involved in phosphotransferase systems (*n* = 19) and ABC-type transporter systems (*n* = 38) were up-regulated only at 40°C and 45°C, while carbohydrate-related metabolism (*n* = 37) and central glycolytic and intermediary pathways (*n* = 29) were enhanced specifically at 45°C. Of the protein functional classes that were consistently repressed at 35°C, 40°C and 45°C, the largest groups of proteins were associated with the terms amino acid-related metabolism (*n* = 46) and nucleic acid/nucleotide metabolism (*n* = 50). Functional classes with mixed responses to thermal stress at different temperatures included proteins associated with DNA repair/recombination (*n* = 16) and co-factor related metabolism (*n* = 28), which were down-regulated at 40°C and 45°C, and proteins involved in lipid-related metabolism (*n* = 27), which overall were attenuated at 45°C specifically.

### Qualitative analysis to reveal proteins specific to culture temperatures

The statistical analysis described above included several proteins that were absent from all three replicates at one specific temperature but detected in all replicates of the three other conditions, and therefore apparently repressed in a temperature-specific manner. From this, we might speculate that proteins that are suppressed, or uniquely expressed, at one or more specific growth temperature(s) could provide insight into survival strategies during prolonged heat stress. Of these proteins, 33 were detected at moderate to high abundance (from average LFQ values) in all the 30°C, 35°C and 40°C CFE samples but not detected at 45°C, and one protein (ThiM) expressed at growth temperatures of 35°C, 40°C and 45°C was not detected at 30°C (Table 2). In addition, based on the complete set of proteins identified in the study, temperature-specific expression patterns were identified in proteins detected in 6/12 samples and 3/12 samples (S4 Table and Table 2). These included proteins detected at 30°C and 35°C only (i.e. apparently suppressed at 40°C and 45°C), notably several proteins involved in purine biosynthesis, and conversely, three proteins that were detected only at 40°C and 45°C. Finally, a group of five proteins was found at one specific temperature only, which included one protein specific to 45°C and detected at moderately high abundance (Asp23_2, alkaline shock protein BN194_01950, average LFQ value ~26) (S2 Table) and four proteins specific to 40°C. The proteins specifically expressed at 40°C and 45°C include proteins involved in sugar, methionine or galactitol uptake and uncharacterized proteins that might participate in α-L-fucosidase activity, glucan-like efflux, toxic substance efflux and general stress responses. The α-L-fucosidase was detected at log_2_-fold 7 higher at 45°C than at 40°C, indicating significantly higher protein expression at this elevated temperature (S2 Table).

**Table 2:** Proteins not detected in CFEs of *Lb*. *casei* GCRL163 for one or more growth temperatures. Proteins were scored as detected (+) when present in all three technical replicates at a specific temperature, or absent (-) when below the level of detection in all three replicates.

Gene name	Protein ID	Gene locus	30°C	35°C	40°C	45°C	Protein names*	Protein functions
*thiM*	K0N1Q6	BN194_03200	-	+	+	+	Hydroxyethylthiazole kinase	Thiamine biosynthesis
*rihC*	K0N803	BN194_03870	-	-	+	+	Non-specific ribonucleoside hydrolase	RNA metabolism, nucleotide salvaging
	K0N8Q2/K0N7Z0	BN194_27750/BN194_27760	-	(+)**	+	+	Putative Alpha-L-fucosidase	Fucose metabolism?
*levE_4*	K0ND69	BN194_27770	-	-	+	+	Fructose-specific phosphotransferase enzyme IIB component	Sugar uptake
	K0MV08	BN194_13440	-	-	+	+	Uncharacterized ABC transporter ATP-binding protein Mb1303c	Lipid export?
*asp23_2*	K0N1F0	BN194_01950	-	-	-	+	Alkaline shock protein 23 family protein	General stress protein
*metN*	K0N4L1	BN194_13750	-	-	+	-	Methionine import ATP-binding protein MetN	Methionine uptake
	K0N6W6	BN194_23110	-	-	+	-	ABC transporter	Toxic substance efflux?
*ppdK*	K0NAC6	BN194_24730	(+)	-	+	-	Pyruvate, phosphate dikinase	Glycolysis
	K0NB06	BN194_27880	-	-	+	-	Autoinducer-2 (AI-2) kinase/class II aldolase	Fucose metabolism?
*tagT*	K0ND99	BN194_27920	-	-	+	-	PTS IIA-like nitrogen-regulatory protein	Transport, galactitol uptake?
	K0N181	BN194_01150	+	+	+	-	Uncharacterized protein	Unknown
	K0N7D4	BN194_01870	+	+	+	-	Uncharacterized protein	Unknown
	K0N5D9	BN194_03130	+	+	+	-	Transcriptional regulator TetR family with acetyltransferase GNAT family domain	Regulation
	K0N281	BN194_04410	+	+	+	-	Uncharacterized protein MJ1651, S-adenosyl-l-methionine hydroxide adenosyltransferase, N- and C-termini	Unknown
*sph*	K0N5Q0	BN194_04930	+	+	+	-	Oleate hydratase	Fatty acid degradation or detoxification?
*mutS2*	K0N359	BN194_08560	+	+	+	-	Endonuclease MutS2	DNA repair (mismatch)
*ung*	K0N758	BN194_11330	+	+	+	-	Uracil-DNA glycosylase	DNA repair (base excision)
*yerH*	K0MUH2	BN194_11940	+	+	+	-	Sex pheromone lipoprotein, CamS superfamily	Unknown
*yfmL*	K0NA13	BN194_12120	+	+	+	-	Putative ATP-dependent RNA helicase yfmL	tRNA/Ribosome assembly/processing
*ycnE*	K0N7R3	BN194_13230	+	+	+	-	Putative monooxygenase, YcnE protein	Unknown
*pepD*	K0N7S0	BN194_13380	+	+	+	-	Probable dipeptidase	Peptidase for amino acid acquisition
*ytsP*	K0N4Q9	BN194_14450	+	+	+	-	YtsP protein	Unknown signal transduction
*ysoA*	K0N4Y3	BN194_15300	+	+	+	-	Uncharacterized protein	Unknown
*hutG*	K0N5I0	BN194_17350	+	+	+	-	N-formylglutamate amidohydrolase	Histidine metabolism
	K0NAY8	BN194_17420	+	+	+	-	Uncharacterized protein, ASCH/PUA domain	Unknown, RNA processing?
*smc*	K0N5H9	BN194_17860	+	+	+	-	Chromosome partition protein Smc	Cell division (chromosomal segegration)
*fmt*	K0N5J2	BN194_18060	+	+	+	-	Methionyl-tRNA formyltransferase	Protein synthesis; translation initiation
*xseA*	K0N5U4	BN194_18200	+	+	+	-	Exodeoxyribonuclease 7 large subunit	DNA repair (mismatch)
*yhaM*	K0NB84	BN194_19070	+	+	+	-	3'-5' Exoribonuclease yhaM	RNA degradation (mRNA turnover?)
	K0N5U0	BN194_19510	+	+	+	-	GNAT family acetyltransferase	Unknown
	K0N5Z4	BN194_20160	+	+	+	-	Uncharacterized protein	Unknown
*oppF_2*	K0MWL1	BN194_20590	+	+	+	-	Oligopeptide transport ATP-binding protein OppF	Oligopeptide uptake
*oppD_2*	K0N6C6	BN194_20600	+	+	+	-	Oligopeptide transport ATP-binding protein OppD	Oligopeptide uptake
*accC*	K0N6M5	BN194_22510	+	+	+	-	Biotin carboxylase	Fatty acid biosynthesis
*fabD*	K0NBT1	BN194_22570	+	+	+	-	Malonyl CoA-acyl carrier protein, Transacylase	fatty acid biosynthesis
	K0N7J8	BN194_23650	+	+	+	-	Amino acid-related metabolism	Unknown
*dnaX*	K0MXD3	BN194_23990	+	+	+	-	DNA polymerase III subunit gamma/tau	DNA replication, elongation
	K0N7R2	BN194_24650	+	+	+	-	YjdJ protein, GNAT family acetyltransferase	Unknown
*pepC*	K0NC50	BN194_24670	+	+	+	-	Aminopeptidase C	Peptidase for amino acid acquisition
*yqiG*	K0NAG4	BN194_25330	+	+	+	-	Probable NADH-dependent flavin oxidoreductase	Unknown
	K0N7Q6	BN194_27210	+	+	+	-	Uncharacterized protein	Unknown
	K0NAX5	BN194_27530	+	+	+	-	Uncharacterized protein	Unknown
	K0MT25	BN194_06540	+	+	-	-	YieF_2 protein, Oxidoreductase activity	Unknown
*purC*	K0N5S8	BN194_19360	+	+	(+)	-	Phosphoribosylaminoimidazole-succinocarboxamide synthase	Purine biosynthesis
*purL*	K0N9H6	BN194_19330	+	+	-	-	Phosphoribosylformylglycinamidine synthase subunit PurL (FGAM synthase) (EC 6.3.5.3)	Purine biosynthesis
*purM*	K0NB95	BN194_19320	+	+	-	-	Phosphoribosylformylglycinamidine cyclo-ligase (EC 6.3.3.1)	Purine biosynthesis
*purQ*	K0MWC7	BN194_19340	+	+	-	-	Phosphoribosylformylglycinamidine synthase subunit PurQ (FGAM synthase) (EC 6.3.5.3)	Purine biosynthesis

* Protein names from UniProt reviewed or submitted names for *Lb*. *casei* W56 or from the RAST SEED finder annotation of *Lb*. *casei* GCRL163 genome [27], with validation by BLASTP in KEGG and NCBI

** Denotes very low LFQ intensity (log_2-_transformed values <<20).

### Expression level of molecular chaperones and other proteins associated with protein misfolding are modulated in *Lb*. *casei* GCRL163 at elevated growth temperature

**Fig 3:**
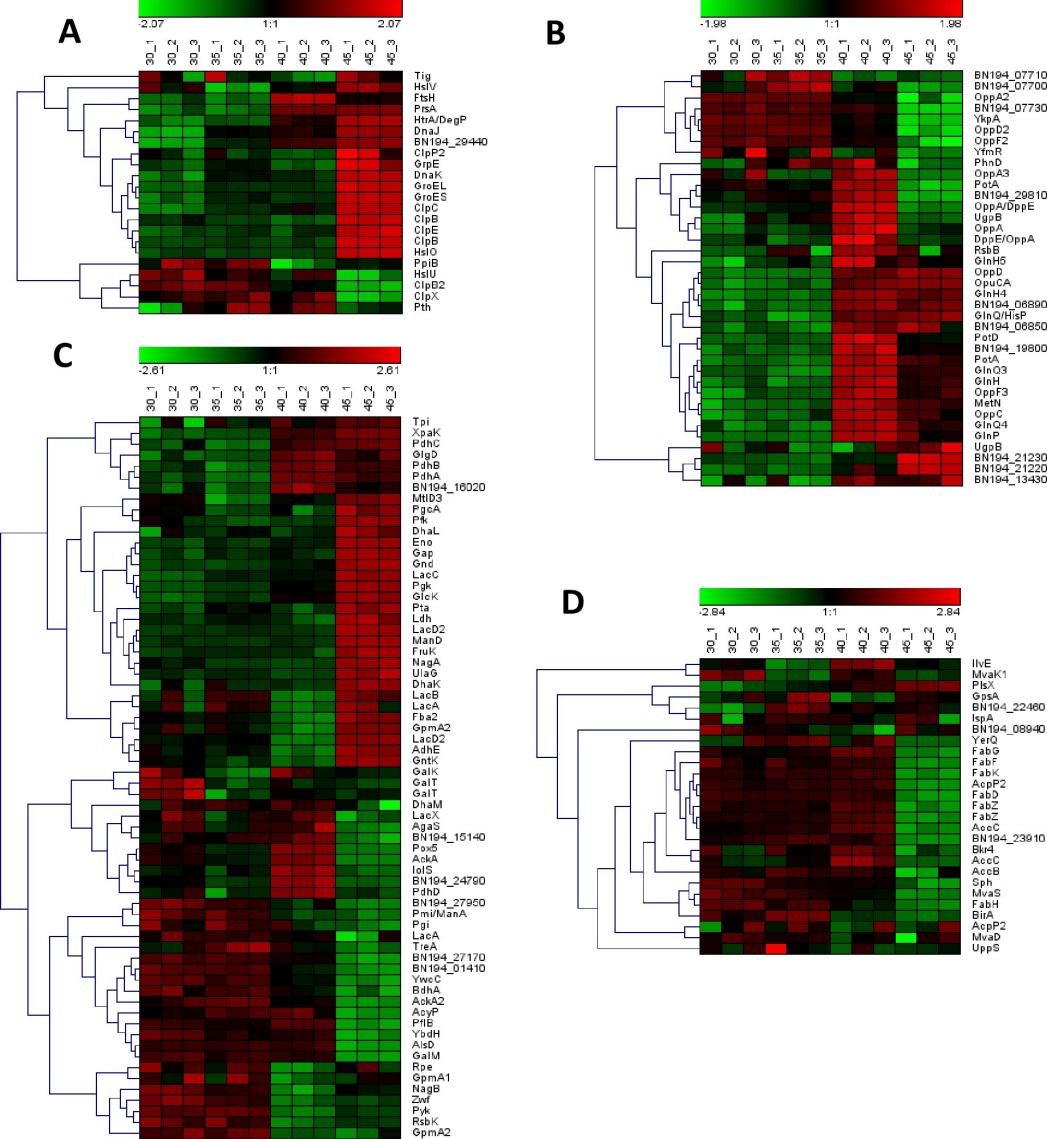
Hierarchical cluster analysis of the abundance of selected proteins in functional classes detected in *Lb*. *casei* GCRL163 CFEs at different temperatures. Mean-centred (Z-scored) LFQ data is shown for triplicates for (A) Protein folding and turnover (B) ABC-type transporter system components (C) Glycolytic/intermediary and carbohydrate metabolism and D) and Fatty acid metabolism functional classes.

The protein functional class ‘protein folding and turnover’ showed higher protein expression levels at all temperatures above 30°C and notably higher overall changes in abundance at 45°C (Fig 2, Fig 3A). However, there were considerable differences in the abundance of the proteins detected and the degree of change in abundance (LFQ values) for individual proteins (Fig 3A, S2 Fig). With the exception of DnaJ and HcrA, the molecular chaperones specified in the GroEL-GroES and HcrA-GrpE-DnaK-DnaJ gene regions, proteins involved in nascent protein folding during normal growth and in heat shock responses in many bacteria [41], were highly abundant proteins at all temperatures. GroEL and GroES were most highly induced at 45°C, by log_2_ 3.6-fold and 3.1-fold, respectively (FDR<1%), with relatively small fold-changes at 35°C and 40°C; DnaK and GrpE followed similar trends. The heat-inducible regulator, HcrA, was detected in low abundance which was not significantly altered in CFEs at different temperatures (S2 Table). DnaJ was also present in low abundance at 30°C and was up-regulated at all temperatures above 30°C (increased log_2_ 1.7-fold, 2.1-fold and 3.3-fold at 35°C, 40°C and 45°C, respectively), showing a different regulatory pattern than the other chaperones. From the published genomes, intergenic regions were detected upstream of the *hrcA*, *grpE*/*dnaK* and *dnaJ* genes in strain GCRL163 and other *Lb*. *casei* strains examined (MJA12, ATCC 334, ATCC 393, W56) and *Lb*. *paracasei* ATCC 25302, although the length of the regions varied in some strains: the BPROM tool detected one promoter in the 112 bp intergenic region upstream of *dnaJ* in all strains except W56. This suggested that the regulation of *dnaJ* may differ between strains and that separate regulation of the genes in this cluster may occur, consistent with the observed proteomic data.

An 18kDa α-crystallin domain heat shock protein (gene locus BN194_29440), which belongs to the small heat shock protein (Hsp20) family, was in very low abundance at 30°C but this was more highly increased with temperature compared with the other heat shock proteins (increased log_2_ 3.1-fold, 5.4-fold and 7.2-fold at 35°C, 40°C and 45°C respectively) (S2 Fig). The degree of upregulation of this protein at temperatures close to the optimum for growth for this strain may suggest a role in inhibiting protein aggregation in response to increases in growth temperature. Other small HSPs (for example Hsp33) were not similarly altered in abundance with increased culture temperature (S2 Fig).

The majority of other heat-inducible proteins, including the Clp family of ATP-dependent proteases, are involved in recycling defective proteins [42]: ClpB, ClpC and ClpE were also strongly induced at 45°C (by log_2_ 2.1-fold, 3.1-fold and 2.2-fold, respectively). Other proteins associated with stabilization or degradation of misfolded/unfolded proteins, such as the foldase PrsA and the serine protease HtrA, were significantly enhanced at both 40°C and 45°C (FDR<1%). With the exception of PepC_2, which was more highly abundant at 40°C (S2 Table), other peptidases were not modulated following growth at different temperatures.

### Expression of proteins in transport systems affected by heat stress

The protein expression data for proteins within the ATP-binding cassette (ABC) transporter systems also revealed a complex pattern of protein modulation across the different temperature treatments, as shown in Fig 3B. A high proportion of these proteins were significantly up-regulated at both 40°C and 45°C relative to 30°C, with maximal expression changes typically detected at 40°C. This applied to several proteins involved in polar amino acid uptake, notably several proteins involved in glutamine uptake including GlnH, GlnH4, GlnQ3 and GlnQ4 (increased log_2_ 2.8-fold, 1.6-fold, 2.3-fold and 1.2-fold, respectively); proteins involved in polyamine uptake such as PotD and PotA_2 (log_2_ 3.5-fold and 2.6-fold increased, respectively); and toxic substance efflux (YfiB, log_2_ 3.3-fold increased). OpuCA, a protein involved in glycine-betaine uptake, and 2 proteins with putative ABC transporter functions (permease, BceA-type transmembrane protein BN194_21220 and ATP-binding protein BceA_2) were most highly induced at 45°C relative to 30°C (increased log_2_ 2.4-fold, 3.9-fold and 3.0-fold, respectively). BN194_21220 also possesses an FtsX-like domain and BN194_21230 an AAA+ ATPase domain [29], which may suggest that these proteins are involved in inserting proteins into cytoplasmic membranes.

At higher growth temperatures, some proteins that facilitate oligopeptide uptake were repressed, whereas others were increased. OppA2, OppD2 and OppF2 were significantly repressed at both 40°C and 45°C relative to 30°C (all FDR<1%, except OppA2 at 45°C, FDR<5%). In contrast, the oligopeptide transport system permease OppC and oligopeptide transport ATP-binding protein OppD were significantly increased at both 40°C and 45°C relative to 30°C (FDR<1% for both proteins at 40°C and FDR<5% for OppC at 45°C) while OppA was elevated at 40°C only (log_2_ 1.84-fold, FDR<1%). These proteins could assist *Lb*. *casei* GCRL163 accumulate oligopeptides from which amino acids are generated through the activity of proteinases including HtrA from degradation of peptidoglycan. Although proteins in the cell wall biogenesis functional class were largely unchanged at higher temperatures in the CFE (see Fig 2), both ManD and NagA, which are involved in degradation of peptidoglycan through the conversion of N-acetyl-glucosamine-6-phosphate into D-glucosamine-6-phosphate thence fructose-6-phosphate plus glutamine (KEGG pathway), were up-regulated at 45°C (Fig 3C), supporting recycling of muropeptides through peptidoglycan turnover [43].

In addition to alterations in amino acid and oligopeptide transporters, most of the proteins included within the functional class ‘phosphotransferase (PTS) system’ were in significantly higher abundance at elevated temperatures (S2 Table). These included FruA3, BglP and SorB_2 which were associated with fructose, β-glucoside and sugar uptake respectively and enhanced at 40°C and 45°C. Other up-regulated PTS proteins are ManY/LevF (log_2_ 2.1-fold at 40°C and 1.2-fold at 45°C), ManY (log_2_ 3.1-fold at 40°C), LevG/ManZ (log_2_ 2.2-fold at 40°C and 1.0-fold at 45°C), ManZ (log_2_ 3.0-fold at 40°C, FDR<1% and 1.6-fold at 45°C, FDR<5%) and LevE/ManX (log_2_ 1.4-fold at 40°C, FDR<5%). However, repression of some PTS proteins was also observed and they included uncharacterized protein BN194_04820 (decreased log_2_ 2.2-fold at 40°C and 45°C) associated with uptake of mannose, fructose or sorbose, MtlA (log_2_ 3.0-fold at 45°C), Lev_3 (log_2_ 2.3-fold at 45°C) and ManX (log_2_ 4.8-fold at 45°C). Lev_4 involved in sugar uptake was specifically expressed at 40°C and 45°C while TagT associated with galactitol uptake was expressed only at 40°C (see Table 2). Several of the PTS proteins are annotated as components of mannose/fructose/sorbose uptake systems but recognised as having broad sugar transport capacity [44] including amino- and nucleotide-sugars. The core PTS proteins, including HprK (increased log_2_ 1.4-fold at 45°C), Ptsl (decreased log_2_ 1.3-fold at 45°C) and PtsH (decreased log_2_ 1.2-fold at 40°C, FDR<5%), are impacted by thermal stress.

### Prolonged heat stress enhanced modulation of proteins involved in carbohydrate metabolism

As a fermentative bacterium when cultured under anaerobic conditions, *Lb*. *casei* GCRL163 must obtain energy by substrate-level phosphorylation [45]. Glucokinase (GlcK), which was enhanced at 40°C (log_2_ 1.1-fold) and 45°C (log_2_ 2.4-fold), might be responsible for the conversion of glucose to glucose 6-phosphate in the bacterial strain with ATP being consumed, during glycolysis (Fig 3C). Similarly, 6-phosphofructokinase catalysing fructose 6-phosphate to fructose 1, 6-biphosphate, which is an energy-consuming step in glycolytic pathway, was enhanced particularly at 45°C (log_2_ 1.3-fold). Several other enzymes associated with glycolysis were modulated during thermal stress, in particular at 45°C. These included phosphofructokinase (Pfk), enolase (Eno), fructose-bisphosphate aldolase (Fba2) and triosephosphate isomerase (Tpi), which were increased log_2_ 1.3-fold, 2.3-fold, 2.5-fold and 1.1-fold, respectively. Glyceraldehyde-3-phosphate dehydrogenase (Gap), which initiates the first substrate-level phosphorylation in glycolysis (through the conversion of glyceraldehyde-3-phosphate to 1,3-bisphosphoglycerate) and phosphoglycerate kinase (Pgk) that dephosphorylates 1,3-bisphosphoglycerate to generate 3-phosphoglycerate and ATP, were significantly up-regulated after growth at 45°C (log_2_ 1.7-fold and log_2_ 2.6-fold respectively). However, Pyk and Fba2 were both repressed at 40°C compared to 30°C.

ATP can be generated through the metabolism of pyruvate to acetate in the pyruvate metabolism pathway [46]. Pyruvate is converted to acetyl-P by Pta and Pox5, then acetyl-P is converted to acetate by AcyP (which is a phosphatase and does not produce energy) and AckA (which does produce energy by substrate level phosphorylation). The majority of enzymes that generate acetate from pyruvate were repressed at 45°C but were more highly abundant at 40°C relative to 30°C (Fig 3C). These included pyruvate oxidase Pox5 (reduced log_2_ 2.0-fold), two acetate kinases AckA and AckA_2 (reduced log_2_ 1.9-fold and log_2_ 1.2-fold, respectively) and the acylphosphatase AcyP (reduced log_2_ 1.7-fold), while Pta and L-Ldh were moderately enhanced (log_2_ 0.9-fold) at the elevated temperature of 45°C. Pyruvate carboxylase and α-oxaloacetate decarboxylase (responsible for the synthesis or degradation of oxaloacetate from pyruvate, respectively), malate dehydrogenase and formate acetyltransferase (pyruvate formate lyase, Pfl) were all inhibited (Fig 3C and S2 Table). Three subunits of the pyruvate dehydrogenase complex (PdhA, PdhB and PdhC) were all increased between log_2_ 1.0-fold to 1.4-fold at elevated temperatures while PdhD was enhanced only at 40°C relative to 30°C. Aldehyde-alcohol dehydrogenase (AdhE) was enhanced at 45°C (log_2_ 3.5-fold, FDR<1%), but repressed at 40°C (log_2_ 2.9-fold, FDR<5%). AdhE can also participate in metabolic pathways involving metabolite biosynthesis, tyrosine and butanoate metabolism, ethanol generation and lipid degradation [30]. Furthermore, the ATP synthase subunits α (AtpA), β (AtpD), b (AtpF), γ (AtpG) and δ (AtpH), associated with membrane bioenergetics, were all up-regulated at 40°C and 45°C. Overall, these results indicate that *Lb*. *casei* GCRL163 induces a complex network of metabolic processes to ensure adequate energy production to meet the physiological demands associated with growth and survival under prolonged heat stress.

### Heat stress repressed expression of proteins associated with fatty acid metabolism

**Fig 4:**
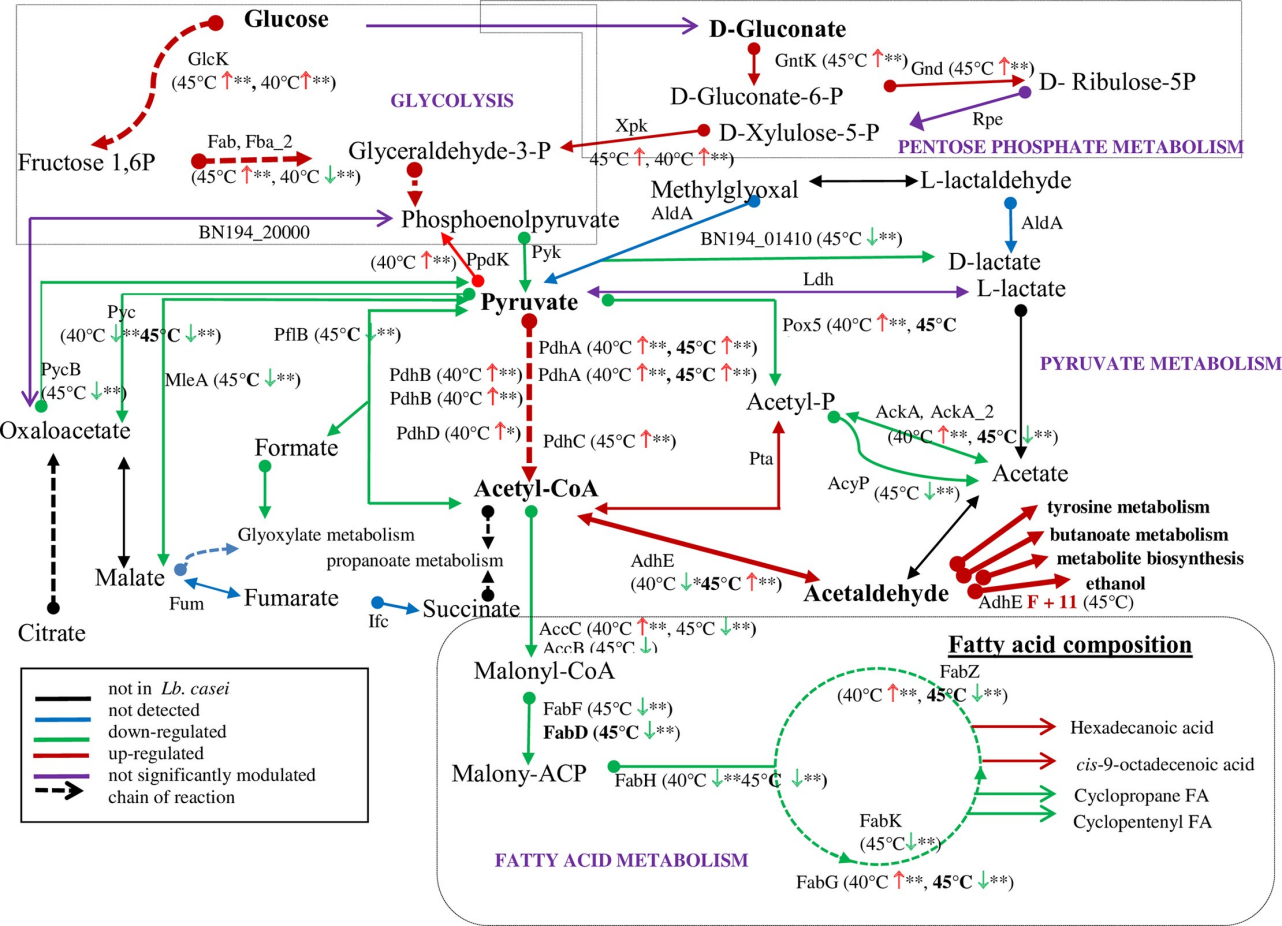
Schematic metabolic map adapted from KEGG pathways showing changes in abundance of some significant proteins in glycolysis, the pentose phosphate pathway, pyruvate and fatty acid metabolism at 40°C and 45°C in relation to 30°C. Coloured arrows in pathways represent changes for cell cultured at 45°C: up- (red) or downregulation (green) or not significantly modulated (purple) or protein not detected (blue) and not known to be present in *Lb*. *casei* (black). Dashed-lines represent a series of reactions, where every enzyme or substrate not is shown. Small arrows (red, up-regulated and green, down-regulated) show changes in abundance at 40°C and 45°C with asterisks indicating FDR (**, FDR<1% and *, FDR<5%). Glck, Glucokinase; Fba, Fructose-bisphosphate aldolase; PfkA, 6-phosphofructokinase; Ldh, Lactate dehydrogenase; Pyc, Pyruvate carboxylase; PycB, Oxaloacetate decarboxylase, alpha subunit; MleA, Malate dehydrogenase; PflB, Formate acetyltransferase/pyruvate formate lyase; PdhABCD, Pyruvate dehydrogenase complex; Pox5, Pyruvate oxidase; AdhE, Aldehyde-alcohol dehydrogenase; Ack, Acetate kinase; AccBC, Acetyl-CoA carboxylase carboxyl transferase complex; FabDFGHK, 3-oxoacyl-ACP synthase complex; Pta, Phosphate acetyltransferase; AcyP, Acylphosphatase; Gnd, 6-phosphogluconate dehydrogenase; XpkA, Xylulose-5-phosphate phosphoketolase and GntK, Gluconokinase.

Proteins involved in lipid-related metabolism that were identified in the current study included enzymes involved in fatty acid biosynthesis that were increased at 40°C, while most were repressed during growth at 45°C (Fig 3D). This could reflect the need to conserve cellular metabolic energy by limiting energy-demanding processes involved in fatty acid biosynthesis. For instance, members of the Fab protein family that were repressed at 45°C included FabD, FabF, FabG, FabH, FabK and two FabZ isoforms (repressed log_2_ 5.4-fold, 1.7-fold, 2.4-fold, 2.1-fold, 1.1-fold, 4.91-fold and 5.3-fold, respectively) while FabG and one FabZ isoform were increased at 40°C (log_2_ 1.1-fold, FDR<5% and log_2_ 1.2-fold, FDR<1%, respectively) (Fig 4). Similarly, the acetyl–CoA carboxylase carboxyl carrier protein AccC was repressed at 45°C (log_2_ 3.3-fold) but enhanced at 40°C (log_2_ 1.6-fold). An AccC isoform (BN194_22500) was also identified to be up-regulated at 40°C (log_2_ 1.6-fold, FDR<5%). Glycerol 3-phosphate acyltransferase PlsX, associated with phospholipid biosynthesis, was the only protein up-regulated at both 40°C and 45°C (log_2_ 1.36-fold, and 2.0-fold, respectively). Oleate hydratase Sph was repressed across the growth temperatures by log_2_ 1.3-fold at 35°C (FDR<5%) and log_2_ 1.6-fold at 40°C and was suppressed beyond detection at 45°C (Table 2). MvaS, involved in isoprenoid biosynthesis in the mevalonate pathway and bifunctional protein BirA were repressed at 40°C and 45°C, while repression of the acyl carrier protein AcpP_2 was observed only at 45°C (log_2_ 3.1-fold).

We also identified cyclopropane fatty acid acyl synthase (BN194_22460), a methyl-transferase putatively involved in the synthesis of cyclic fatty acid cyclopropane (which is in gene neighbourhood of the fatty acid synthesis genes), that was most highly induced at 35°C (log_2_ 1.1-fold, FDR<5%) with lesser increases at 40°C and 45°C (log_2_ ~0.7-fold, FDR<5%). Since changes in membrane fatty acid composition have been associated with heat stress in lactobacilli [47], we therefore investigated the relative percentages of the cell fatty acids with respect to growth phase and temperature of growth (Table 3).

**Table 3:** Fatty acid composition of *Lb*. *casei* GCRL163 cells harvested across the growth cycle when cultured at 30 to 45°C.

Fatty acids			30°C			35°C			40°C			45°C		
Systematic names	Common names	FA designation	ML^a^	LL	ST	ML	LL	ST	ML	LL	ST	ML	LL	ST
*cis*-7-Tetradecenoic acid		14:1(n-7)c	0.1 ^b^	0.2	0.2	0.3	0.2	0.2	0.2	0.2	0.1	0.0	0.0	0.0
Tetradecanoic acid	Myristic acid	14:0	3.3	3.9	3.1	3.7	2.1	2.5	3.3	2.8	3.2	5.1	3.9	4.8
13-Methyltetradecanoic acid	*iso*-Pentadecanoic acid	i15:0	0.1	0.0	0.0	0.0	0.0	0.0	0.0	0.0	0.0	0.0	0.0	0.2
12-Methyltetradecanoic acid	*anteiso*-Pentadecanoic acid	a15:0	0.2	0.1	0.1	0.1	0.0	0.1	0.1	0.1	0.1	0.0	0.0	0.0
Pentadecanoic acid	Pentadecylic acid	15:0	0.2	0.0	0.0	0.1	0.0	0.0	0.1	0.1	0.0	0.0	0.3	0.3
*cis*-9-Hexadecenoic acid	Palmitoleic acid	16:1(n-7)c	4.3	4.4	3.5	3.3	3.2	4.0	3.5	3.2	2.9	4.5	3.6	3.0
*trans*-9-Hexadecenoic acid		16:1(n-7)t	0.2	2.8	2.2	0.1	2.8	3.2	0.7	2.8	2.7	0.0	0.0	0.0
*cis*-11-Hexadecenoic acid		16:1(n-5)	0.5	0.6	0.5	0.3	0.5	0.5	0.3	0.5	0.5	0.0	0.0	0.0
Hexadecanoic acid	Palmitic acid	16:0	15.1	22.3	20.7	10.4	27.0	24.6	10.7	25.5	27.2	26.4	22.1	21.4
		*cyclopentenyl-16:0	0.0	0.4	0.2	0.0	0.4	0.7	0.2	0.4	0.3	0.0	0.0	0.0
14-Methylheptadecanoic acid	*anteiso*-Heptadecanoic acid	a17:0	0.3	0.1	0.1	0.2	0.1	0.1	0.1	0.1	0.1	0.0	0.0	0.4
*cis*-9-Heptadecenoic acid	Margarolic acid	17:1(n-8)	0.7	0.2	0.2	0.4	0.1	0.1	0.3	0.1	0.2	0.0	0.5	0.3
Heptadecanoic acid	Margarinic acid	17:0	0.1	0.0	0.0	0.0	0.0	0.0	0.0	0.0	0.0	0.0	0.1	0.0
*cis*-9-Octadecenoic acid	Oleic acid	18:1(n-9)	66.4	29.2	33.0	67.7	21.8	17.6	58.7	23.9	20.2	50.2	49.6	55.1
*cis*-11-Octadecenoic acid	Vaccenic acid	18:1(n-7)	2.9	28.3	29.0	3.1	32.2	30.0	8.3	25.6	23.1	2.0	4.0	2.3
Octadecanoic acid	Stearic acid	18:0	0.9	1.1	1.2	1.8	1.8	1.8	1.3	2.0	2.0	0.9	1.0	1.1
		*cyclopentenyl-18:0a	0.5	1.4	0.7	1.3	0.6	0.5	1.5	1.0	0.8	0.0	0.4	0.0
		*cyclopentenyl-18:0b	0.4	0.9	0.8	1.3	0.6	0.6	1.3	0.6	0.8	0.0	0.4	0.5
		*cyclopentenyl-18:0c	0.0	0.0	0.1	0.2	0.1	0.0	0.0	0.0	0.1	0.0	0.0	0.0
		*cyclopentenyl-18:0d	0.6	0.8	0.8	1.2	0.5	0.5	1.3	1.0	0.9	0.0	0.5	0.5
		*cyclopropane-18:0a	2.7	2.1	2.3	4.5	3.2	6.5	7.5	7.3	9.2	10.9	13.5	10.2
		*cyclopropane-18:0b	0.5	1.4	1.3	0.0	2.7	6.5	0.4	2.7	5.4	0.0	0.0	0.0
		Total	100	100	100	100	100	100	100	100	100	100	100	100

^a^ Growth phases: ML, mid-log; LL, late log; ST, stationary

^b^ Fatty acid composition is expressed as the percentage of each fatty acid relative to the total amount detected in an extract. The figures represent two independent experiments conducted.

*Full systematic names could not be assigned as full structures were not determined.

### Impact of prolonged heat stress on fatty acid composition

In this study, the principal unsaturated fatty acid was *cis*-9-octadecenoic acid (oleic acid) while the dominant saturated fatty acid was hexadecanoic acid (palmitic acid). Formation of palmitic acid increased from exponential to stationary phases in cells grown at 30 to 40°C (Table 3). Furthermore, in mid-exponential phase, the proportion of palmitic acid increased from 15.1% at 30°C to 26.4% at 45°C. The proportion of oleic acid detected decreased as the cells aged from exponential to stationary phases and also with increase in growth temperature. At 30°C, the proportion of oleic acid decreased from 66.4% at mid-exponential phase to 33.0% at stationary phase was observed, with concomitant increases in vaccenic acid. Similar trends were noted in cells grown at 35°C and 40°C across the growth phases. Reduction in the proportion of oleic acid from 66.4% at 30°C to 50.2% at 45°C in mid-exponential phase was also observed. Synthesis of *cis*-11-octadecenoic acid (vaccenic acid) was observed to be growth phase- and temperature-dependent, as production increased from exponential to stationary phase and across the growth temperatures. Two isomers of cyclopropane FA (cyclopropane-18:0a and cyclopropane-18:0b) and novel cyclopentenyl moieties, four isomers of C18:0 (retention index values of 2144, isomer a; 2153, isomer b; 2170, isomer c and 2187 isomer d), formed which are likely products of oleic acid cyclization in the cells. Production of the cyclopentenyl moieties was, however, reduced at 45°C. Our data demonstrate that the phase of growth and culturing temperatures substantially modulate FA composition in *Lb*. *casei* GCRL163 and the cells are conserving energy by adjusting the existing fatty acids rather than synthesizing new ones.

### Prolonged heat stress enhanced post-translational protein secretion systems and thiamine metabolism

**Fig 5:**
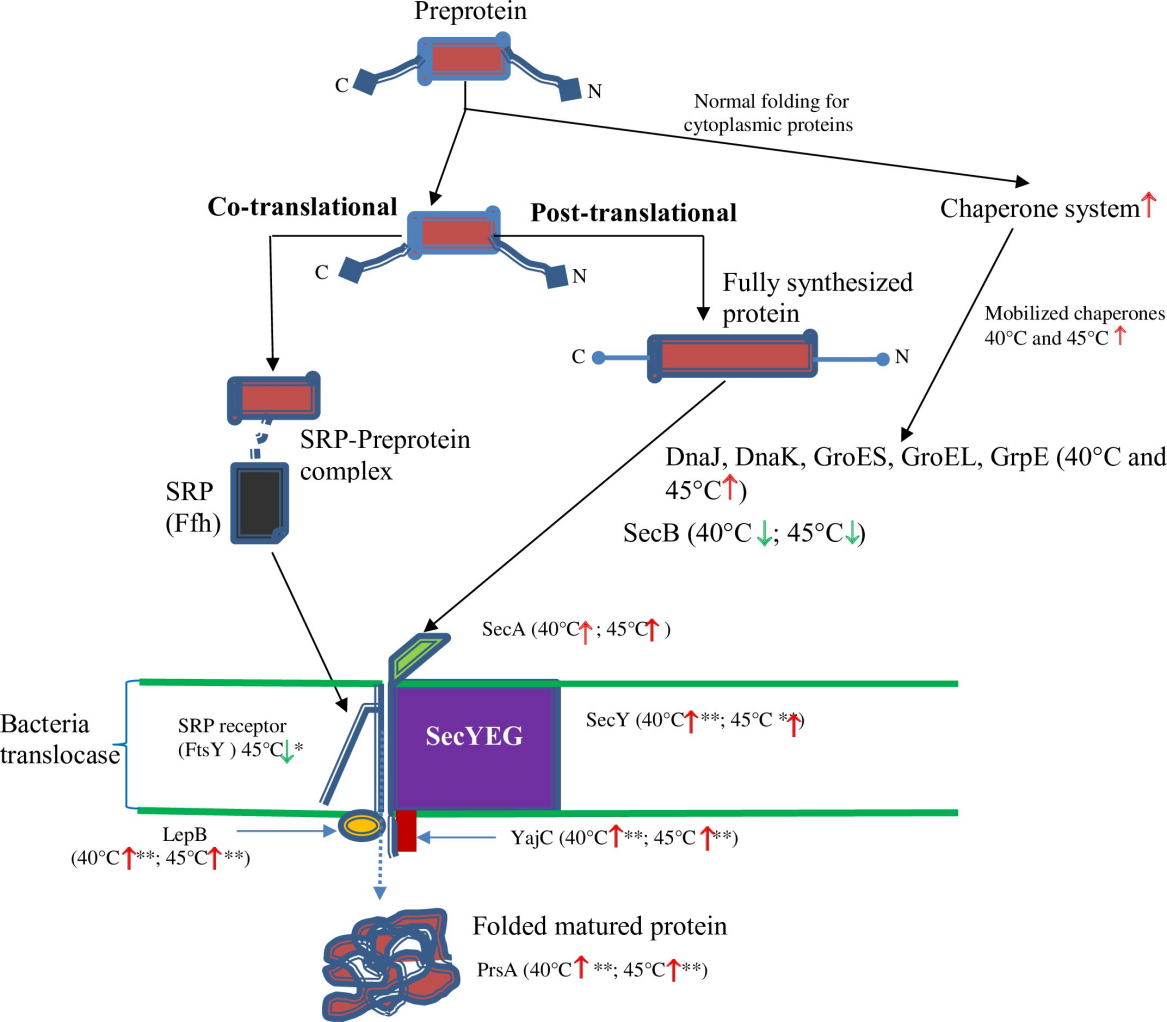
Proposed schematic representation of the components of *Lb*. *casei* GCRL163 translocase system showing proteomic changes under prolonged heat stress. Ffh, signal recognition particle (SRP) protein; FtsY, signal recognition particle receptor; LepB, signal peptidase I; YajC, preprotein translocase insertion/stabilisation protein; SecY, preprotein translocase apparatus protein; SecA, Protein translocase subunit SecA; SecB, Uncharacterized BN194_10000; PrsA, foldase protein; DnaJ, chaperone protein DnaJ; GroEL, class I heat-shock protein (chaperonin) large subunit; GrpE, molecular chaperone (heat shock protein, Hsp70 complex). Up-regulated proteins are indicated by red and down-regulated by green arrows with asterisk indicating FDR (**, FDR<1% and *, FDR<5%).

Proteins associated with post-translational protein secretion belonged to a relatively small but important subgroup of proteins that were differentially expressed during prolonged heat stress (Fig 5). The cytoplasmic ATPase SecA, reported to channel precursor proteins into the SecYEG translocon channel used for preprotein translocation across the plasma membrane, and YajC encoding protein that participates in liberating the preproteins from the channel [48] were both enhanced at 40°C and 45°C, and the central *S*ecY subunit itself was detected at increased levels at 40°C (log_2_ 3.0-fold; FDR>5%) and 45°C (log_2_ 3.2-fold; FDR<5%). Upregulation of the signal peptidase LepB at both 40°C and 45°C (log_2_ 1.8-fold and 1.9-fold, respectively; FDR<1%) was consistent with a role for LepB in the release of mature polypeptides [49] from the channel under thermal stress. Several chaperones contribute to post-translational secretion in Gram-positive and Gram-negative bacteria, such as SecB [50], heat shock proteins DnaK, DnaJ and GrpE [51] and also trigger factor Tig (peptidyl-prolyl isomerase) [52]. In our current study, we identified an uncharacterized 15 kDa protein with gene locus BN194_10000 with the domain of export chaperone SecB in *Lb*. *casei* GCRL163, which is conserved in several genomes of *Lb*. *casei* group strains as documented on UniProt [29]. This protein was maximally expressed at the control temperature of 30°C and significantly repressed at 40°C and 45°C (by log_2_ 2.3-fold and 4.1-fold, respectively) in *Lb*. *casei* GCRL163. Trigger factor Tig, which is a protein export chaperone that ensures newly synthesized proteins are kept in an open conformation, was not significantly induced during prolonged heat stress but was detected in in CFEs at very high abundance (S2 Table). Up-regulated foldase protein PrsA at elevated temperature is a chaperone protein associated with post-translocational folding of many secreted proteins extracellularly. These results suggest that increased secretion, especially through the post-translational system, may be one of the major changes involved in the survival of *Lb*. *casei* GCRL163 during prolonged heat stress.

Our proteomic data (S2 Table) further revealed that most proteins associated with cofactor-related metabolism were downregulated at 40°C and 45°C including proteins encoded by LplJ, PncB, SufB, SufC, FolA, ApbE and IscS. However, in stark contrast, the hydroxyethylthiazole kinase ThiM, which is associated with thiamine biosynthesis, was the most highly up-regulated protein at all three elevated temperatures (log_2_ 4.1-fold at 35°C, FDR<5%; 6.6-fold at 40°C and 7.3 at 45°C) (Fig 1, Table 1). The biologically active derivative of thiamine, thiamine diphosphate, is an important cofactor for several enzymes associated with amino acid and carbohydrate metabolism [53].

### The *Lb*. *casei* SecB homolog is phylogenetically distant from *E*. *coli* SecB but structurally similar

BLASTP (KEGG) of the *Lb*. *casei* GCRL163 SecB homolog (protein sequence from the RAST file of the sequenced genome [27] showed 100% sequence similarity with *Lb*. *casei* and *Lb*. *paracasei* proteins while *Lb*. *rhamnosus* strains were 91.9–92.6% similar to the *Lb*. *casei* GCRL163 SecB homolog sequence (S3 Fig). Although the *Lb*. *casei* and *Lb*. *rhamnosus* proteins were closely related and conserved across the strains reviewed, they formed separate clades and were distinct from the other *Lactobacillus* species. Other proteins with SecB domains were identified in UniProt searches for ‘SecB’ then by genus (*Escherichia*, *Bacillus*, *Lactobacillus*), and a selection of these were chosen for sequence similarity analysis: a reviewed entry representative of Gram-negative SecB proteins (*E*. *coli* K12 protein ID P0AG86, where all top 50 hits for *E*. *coli* were 100% identical to this protein); two proteins from Gram-positive species with a submitted but unreviewed name of ‘Preprotein translocase subunit SecB’ (*Bacillus* sp. CC120222-01 protein A0A1X7ETD2 and *Lb*. *plantarum* subsp. *plantarum* ATCC14917 protein D7V892); and an uncharacterised *Bacillus* protein with a domain structure similar to the *Lb*. *casei* domains (*Bacillus licheniformis* CG-B52 uncharacterised protein T5HC28, with overlapping homologous domains DUF1149, SecB [IPR003708] and SecB-like superfamily [IPR035958]) (S3 Fig). The presumptive SecB detected in *Lb*. *casei* GCRL163 showed low sequence similarity with *E*. *coli* SecB (16.0%) whereas the *Bacillus* and, interestingly, *Lactobacillus plantarum* subsp. *plantarum* orthologs, showed greater similarity (48–54%).

**Fig 6:**
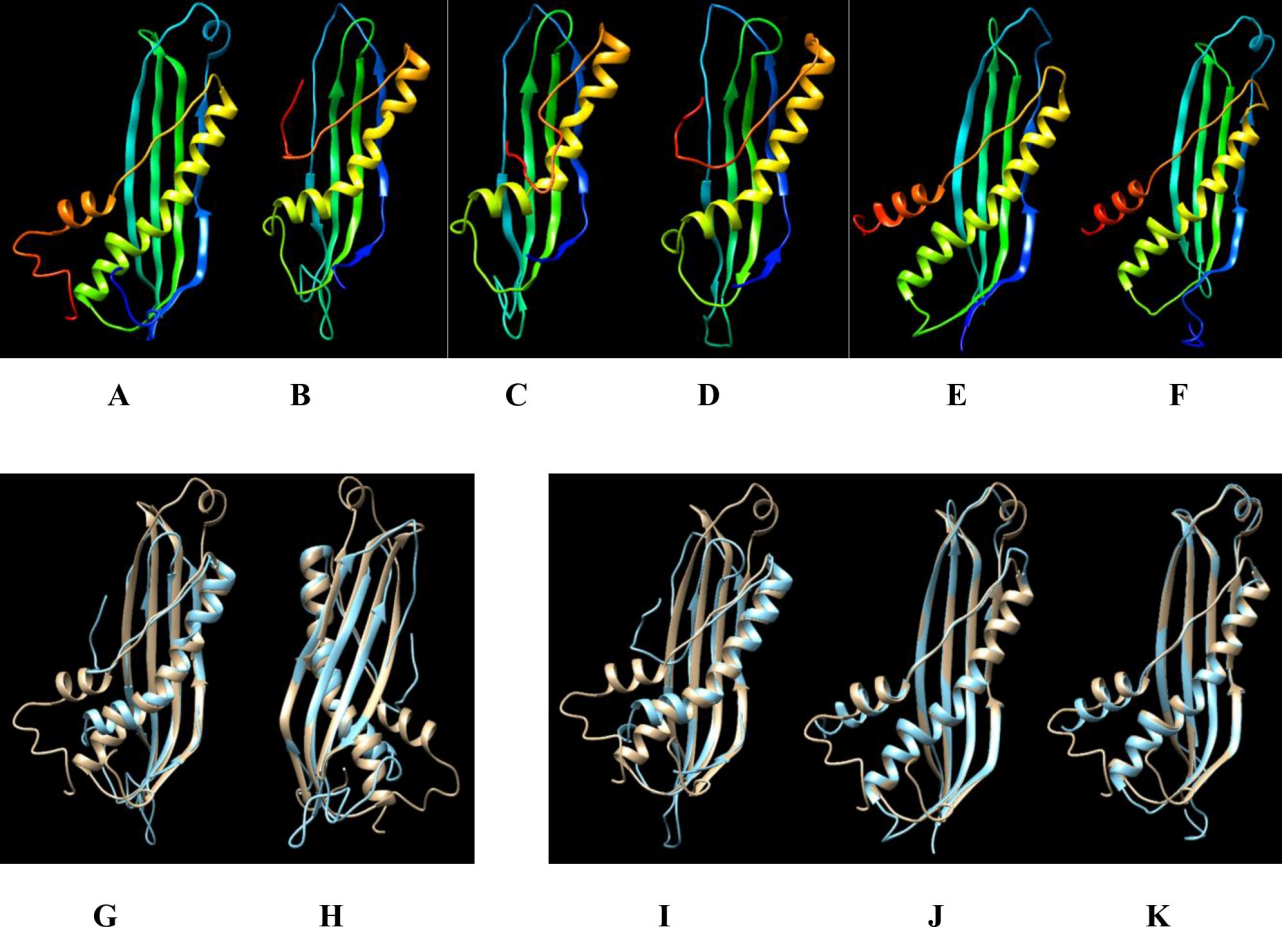
Modelled 3D-structures of proteins annotated as SecB and homologs with SecB-like superfamily domains (IPR035958) and structural alignment with an *E*. *coli* SecB. Proteins were modelled using Phyre2 and aligned using UCSF Chimera software. Protein identities (UniProt or NCBI) and lengths (amino acids) are: **A**, *E*. *coli* K12 SecB P0AG86 (155); **B**, *Lb*. *casei* GCRL163, uncharacterised protein (137); **C**, *Lb*. *casei* W56, uncharacterised protein K0N3D6 (gene locus BN194_10000) (136); **D**, *Lb*. *rhamnosus* GG, conserved protein CAR86774 (gene locus LGG_00879) (135); **E**, *Lb*. *plantarum* subsp. *plantarum* ATCC14917 SecB D7V892 (142); **F**, *B*. *licheniformis* CG-B52, uncharacterised protein A0A2234XY3 (138). Panels **G** to **K** align the *E*. *coli* SecB P0AG86 template (gold) with the proteins from the following species (blue): **G**, *Lb*. *casei* GCRL163, α1-helices central; **H**, *Lb*. *casei* GCRL163, view of β-strands; **I**, *Lb*. *rhamnosus*; **J**, *Lb*. *plantarum* subsp. *plantarum*; **K**, *B*. *licheniformis*. Ribbon colour: rainbow N to C terminal (red).

X-ray analysis of crystal structures of *E*. *coli* and *Haemophilus influenzae* SecB proteins [54] show a common structure consisting of 4 anti-parallel β-sheets (β1 and β2 are outer strands), a main α1-helix and C-terminal anti-parallel α2-helix and a crossover loop between β1 and β2, all of which have proposed functional roles in binding client proteins or binding to SecA [55]. The 3D protein models for *E*. *coli*, *Lb*. *plantarum* subsp. *plantarum* and *B*. *licheniformis* exhibit these common structural features (Fig 6A, 6E and 6F), supporting the contention that the uncharacterised proteins in the latter two species are functionally similar. However, the *Lb*. *casei* (W56, GCRL163) and *Lb*. *rhamnosus* GG models (Fig 6B, 6C and 6D) lack the second C-terminal helix (although this region on all of the models has a low confidence score), and also possess a truncated β2-strand and a diminished crossover loop. Notwithstanding these differences, and the difference in protein sizes, when aligned against the *E*. *coli* protein (Fig 6G–6K), the overall architectures of all of the proteins are similar.

## Discussion

Stress physiology of LAB has been reviewed extensively and, despite genomic differences and strain-to-strain variation in responses across and within species, and differences in experimental approaches employed, there are striking similarities in how this group of genera responds to stressors [6, 13, 14]. Common global responses include protection of macromolecules involved in metabolic activities (notably through chaperones engaged in protein folding and turnover) or cellular structural integrity (cell envelope components, including membrane lipids); modulation of metabolic activity and energy production to ameliorate the impact of stress, typically around the fate of pyruvate; altered uptake of alternative carbon sources to facilitate increased energy production; and increased proteolysis and catabolism of amino acids. While growth in bioreactors at 40°C was marginally impaired, 45°C caused significant decline in growth rate and rapid entry into stationary phase. The proteomes for these two temperatures were also markedly different to cells cultured at lower temperatures and to each other, while sharing many common trends with the global responses documented for LAB, particularly in greater abundance of ABC- and PTS-transporters plus rerouting carbohydrate metabolism. Many of the proteins with highly altered abundance, or unique detection, at 45°C were functionally uncharacterized but domain structures detected and phylogenetic analyses infer presumptive activity of proteins which have not previously been documented in heat stress responses in lactobacilli.

Chaperones GroES, GroEL, DnaK and GrpE were up-regulated significantly only at 45°C, consistent with many previous reports for heat shock responses in LAB, while DnaJ was increasingly more abundant with temperature. Although the gene cluster including the *hrcA* heat inducible regulator, *grpE*, *dnaK* and *dnaJ* are considered to form an operon which is conserved across *Lactobacillus* species [8], our proteomic data indicated separate regulation of these genes as growth temperature increased, an observation supported by detecting promoters in intergenic regions, which has not been reported for heat shock responses in LAB. Expression of the chaperone systems during prolonged heat stress was more enhanced than the proteases, suggesting that protein refolding and recycling were preferred to proteolysis as a mechanism for protein quality control in *Lb*. *casei* GCRL163. A novel observation from our data was the consistent increase in abundance with temperature of an α-crystallin domain protein encoded by gene locus BN194_29440 and annotated as an acid shock protein [29], was more highly up-regulated in response to heat stress compared to all other molecular chaperones. The α-crystallin heat shock proteins possess a conserved central α-crystallin domain region of about 80 amino acids and form part of the highly synergistic multi-chaperone network induced usually under stress conditions and weakly under normal conditions in cells [56]. The ATP-independent chaperone activity is limited to the prevention of irreversible protein aggregation by binding to unfolding intermediates and may pay a role in cell surface integrity [56]. The expression of the α-crystallin heat shock protein family is restricted to some members of the eukaryotes, archaea and bacteria, and is absent, for example, in *Mycoplasma genitaliun* [57] and *Helicobacter pylori* [58]. In LAB, including *Lb*. *delbrueckii*, *Lb*. *helveticus*, *Oenococcus oeni* and *S*. *thermophilus*, small heat shock proteins related to eukaryotic α-crystallins have been reported [59, 60]. Interestingly, despite the extensive literature on the mechanism of the highly-conserved chaperone proteins in protein folding in numerous bacterial species, Papadimitriou *et al*. (2016) noted that studies on the mechanisms of action of chaperones in LAB are lacking [6]. This suggests that there is scope for further investigations on the role of small HSPs, particularly in context of the greater expression of the Hsp20 protein as culture temperature increased (reported in the current study) and prior reports on stress-induced small HSPs in *Lb*. *plantarum* and *O*. *oeni* [6].

Our current investigation further provides findings on the involvement of the chaperone systems in the post-translational secretion in *Lb*. *casei* GCRL163 during prolonged heat stress, with the BN194_10000 protein, consisting of 136 amino acids and with a superfamily domain structure consistent with a protein export chaperone SecB, identified. The translocation of proteins across the membrane is thought to be facilitated by the chaperone function of the heat shock proteins in bacteria and eukaryotes [61, 62]. In *E*. *coli*, secretion of outer membrane proteins occurs principally through the SecB-dependent pathway with SecB binding to non-native proteins and presenting them in competent state to SecA in the Sec translocon [63, 64]. However, in SecB deficient strains, DnaK, DnaJ, GroES-EL and GrpE are the main chaperones [51, 62]. SecB is essential for protein folding in *E*. *coli* at low temperatures and was shown to compete with the GroES-EL and DnaK-DnaJ-GrpE chaperones for unfolded proteins: as growth temperature increases, SecB is repressed while chaperones are induced [65]. Few SecB homologs in Gram-positive bacteria have been linked experimentally with preventing protein folding and aggregation [66, 67] and most of the current knowledge concerning the Sec pathway is based on studies in *E*. *coli* and other Gram-negative species. The exceptions are the CsaA protein in *B*. *subtilis* (110 amino acids) [68] and the SecB-like protein of *Mycobacterium tuberculosis* (181 amino acids) [68, 69], which are not structurally similar to the Gram-negative SecB proteins but are able to complement *E*. *coli* SecB mutants [68, 70]. A phylogenetic analysis of 3,813 SecB genes, identified in UniProt in bacteria with full taxonomic lineage and across all phyla, showed that the sequence similarity between genes was often very low but the protein architecture is preserved despite differences in protein size (mean of 157 ±27 amino acids) [71]. The modelled 3D-structures of *Lb*. *casei* GCRL163 and *Lb*. *rhamnosus* SecB homolog proteins showed architecture generally similar to the SecB of *E*. *coli*, and close structural alignment between the proteins was evident. However, this structure differed from the *E*.*coli*, *Lb*. *plantarum* and *B*. *licheniformis* model structures, notably the absence of the α2, C-terminal helix. Although the structure of the SecB protein is important for forming a tetramer which is a ‘dimer of dimers’ [55], prior studies had shown that deletion of the α2-helix did not alter client protein binding but did debilitate interactions with SecA [54]. It remains to be demonstrated whether the arrangement of residues in the presumptive *Lb*. *casei* SecB protein supports dimer plus tetramer formation and whether the structure supports client protein binding. Evidence for the proposed function of the SecB homolog in *Lb*. *casei* in the current study is based on global architectural similarity and the observed expression pattern of the protein during culture at temperatures below optimum. A reasonable first step in demonstrating functionality would be determining whether expression of the *Lb*. *casei secB* complements knock-out mutants of *E*. *coli*, similar to the studies which demonstrated the chaperone role of CsaA in *B*. *subtilis*. To our knowledge, this is the first report of a SecB homolog in lactobacilli, which are generally thought to lack SecB [72].

In bacteria, preproteins with N-terminal, hydrophobic core and C-terminal domains can be translocated through co-translational system by binding to the signal recognition particle (SRP) Ffh protein [73]. Although the abundance of Ffh was not modulated by heat stress, several components of the Sec secretion pathway (SecA, which channels the preprotein to SecYEG and translocase subunit SecY), proteins YajC and LepB (responsible for the release of preprotein and matured protein from SecYEG channel) and PrsA were more abundant at 40 and 45°C. Greater abundance of these proteins suggests that greater levels of protein secretion to or through the cell surface may occur during prolonged heat stress. Despite the importance of the surface and extracellular proteome of lactobacilli in probiotic function, there is relatively little reported on the impact of stress on the secretome [72] with the exception of recent studies on S-layer producing species [74]. Examining the surface and secreted proteins following culture at supra-optimal temperatures of *Lb*. *casei* GCRL163 will expand our knowledge on the yet uncharacterized heat stress responses of lactobacilli.

As observed for other stress responses in LAB [6, 13], the expression of proteins around pyruvate metabolism was impacted by growth at 40 and 45°C. However, the pattern of expression differed at the two temperatures: at 40°C, the anticipated upregulation of proteins associated with pyruvate conversion to acetyl-CoA thence acetate was observed [6], whereas at 45°C, the abundance of the Pdh complex subunits involved in the formation of acetyl-CoA was similarly enhanced while all other proteins associated with formation of acetate, with the exception of Pta, were repressed. A key difference at 45°C was the large increase in abundance of AdhE, which has not been reported previously for *Lb*. *casei* or other LAB. The fate of acetyl-CoA was not experimentally explored in this study, but the pattern of protein expression suggests altered carbon flux to acetate with possible accumulation of acetyl-P or formation of acetaldehyde or ethanol end products and lowered energy production through conversion of acetyl-P to acetate at 45°C. In acid stress, pyruvate metabolism is rerouted into the synthesis of fatty acid synthesis in *Lb*. *delbrueckii* subsp. *bulgaricus* and *Lb*. *rhamnosus* [75, 76], a route which is not taken in prolonged heat stress given the decline in expression of fatty acid synthesis proteins observed at 45°C. Furthermore, all of the ATP synthase subunits were up-regulated at 40°C and more so at 45°C. These proteins are linked to the maintenance of intracellular pH through proton extrusion by the F_0_F_1_-ATPase and maintenance of a PMF by the membrane bound F_0_F_1_-ATP [46, 77]. Our data demonstrate that under prolonged heat stress cellular energy is sourced more through ATP synthase activity in this strain.

Modulation of some glycolytic pathway proteins is an important mechanism in LAB for generating energy in the form of ATP during heat and other stresses [7, 78, 79], although the patterns of modulation vary with genera and stress type [6]. It can be inferred that *Lb*. *casei* GCRL163 cells consume more energy at elevated temperature, which is consistent with ATP usage by chaperones at higher temperature [80]. The bacteria therefore establish a network of mechanisms to generate energy for survival during thermal stress but are very conservative in the energy-dispensing processes thereby shutting down high energy-requiring pathways, such as fatty acid biosynthesis, especially at 45°C. Our data suggested that oleic acid was the principal unsaturated fatty acid at this temperature with vaccenic acid greatly reduced, at all growth phases. At other temperatures, oleic acid decreased from exponential to stationary phase. This temperature-dependent reduction in the degree of fatty acid unsaturation was also observed in *Lb*. *fermentum* as a result of temperature-dependent shift between vaccenic to oleic acid below 20°C and above 26°C with oleic acid cyclized to cyclopropane acids by cyclopropane synthetase [81]. The synthesis of vaccenic and oleic acids is not well defined under anaerobic conditions although it is hypothesised this occurs by insertion of a double bond during chain elongation by the action of the Fab enzymes [81, 82]. Decrease in vaccenic acid production may indicate either temperature-sensitive impairment of insertion of a double bond during chain elongation (together with reduction of fatty acid synthesis enzymes) or failure to subsequently epimerise oleic into vaccenic acid. This remains a subject for further investigation.

In *Lb*. *casei* GCRL163, expression of cyclopropane fatty acid acyl synthase which could be responsible for the cyclization of oleic acid to cyclopropane was up-regulated maximally at 35°C with only minor increases at 40°C and 45°C relative to 30°C. Our GC/MS data also revealed novel cyclopentenyl moieties produced in all the growth phases which might have been synthesized from the cyclization of oleic acid with decreased synthesis at 45°C. Cyclopentenyl fatty acids have previously been reported in plants [83] and oil [84] but not in bacteria. To the best of our knowledge this represents the first report of the cyclopentenyl moieties in LAB. Fatty acid biosynthesis is an energy intensive process and cells have the capacity to adjust existing fatty acids thereby conserving energy that would be otherwise spent in synthesising new fatty acids [85], which we suggest occurs during growth at 45°C.

Several other proteins related to DNA repair and recombination, posttranslational modification, amino acid metabolism, nucleic acid and nucleotide metabolism were also repressed at higher growth temperatures. Indeed, the pattern of protein expression for cells cultured at 45°C (repression of DnaA, ribosome content and proteins associated with purine biosynthesis) is reminiscent of transition into stationary phase [86, 87], which is consistent with the growth kinetics observed. However, greater abundance of proteins involved in sugar uptake, the tagatose-phosphate pathway (Lac proteins) and greater expression of an α-L-fucosidase at 45°C, plus upregulation of proteins involved in forming fructose-6-phosphate (ManD, NagA), suggest both carbon source scavenging and possible turnover of peptidoglycan. The unique detection of Asp23_2 at 45°C may also indicate cell surface structural changes beyond membrane lipid content, given the documented role of this class of stress protein in cell wall stress in *Staphylococcus aureus* [88] and the recent implication of this protein in cell surface changes in gentamycin resistant *Lb*. *casei* [18]. Our study of proteomic changes during growth at inhibitory, supra-optimal temperatures has revealed new findings on how *Lb*. *casei* GCRL163 responds to prolonged heat stress and opens up further avenues for investigation on the network of physiological changes required to enhance cell survival.

## Supporting information

S1 Table MaxQuant output files *peptides*.*txt* and *proteinGroups*.*txt* that summarise the complete sets of peptides and proteins, respectively, identified in *Lb*. *casei* GCRL163 samples. (XLSX)

S2 Table Proteins that meet the filtering criteria and statistical comparison of LFQ protein expression data for *Lb*. *casei* GCRL163 proteins at different heat adaptations. (XLSX)

S3 Table Mean-centred (Z-scored) LFQ expression data and functional annotation of *Lb*. *casei* GCRL163 proteins based on the result of T-profiler analysis. (XLSX)

S4 Table Filtered proteins based on proteins identified in at least 3 replicates, to identify proteins specific to temperatures in *Lb*. *casei* GCRL163. (XLSX)

S1 Fig Maximum specific growth rates and growth curve of *Lb*. *casei* GCRL163 at different temperatures. (PDF)

S2 Fig Abundance of selected chaperone proteins in triplicate samples of CFEs for cells harvested at mid-exponential growth at temperatures from 30 to 45°C. (PDF)

S3 Fig Sequence alignment for selected SecB proteins using Clustal Omega (multiple-sequence) and Needle (pair-wise comparisons) (EMBL-EBI, www.ebi.ac.uk) and cladogram constructed in the EMBL-EBI tools suite. (PDF)
