# Supplementary material for: Proteomic analysis of Lactobacillus casei GCRL163 cell-free extracts reveals a SecB homolog and other biomarkers of prolonged heat stress
Source: PLoS One. 2018 Oct 25;13(10):e0206317. doi: 10.1371/journal.pone.0206317 (PMC6201924; doi:10.1371/journal.pone.0206317)
Supplement: S1 Fig — (PDF) [file pone.0206317.s005.pdf]

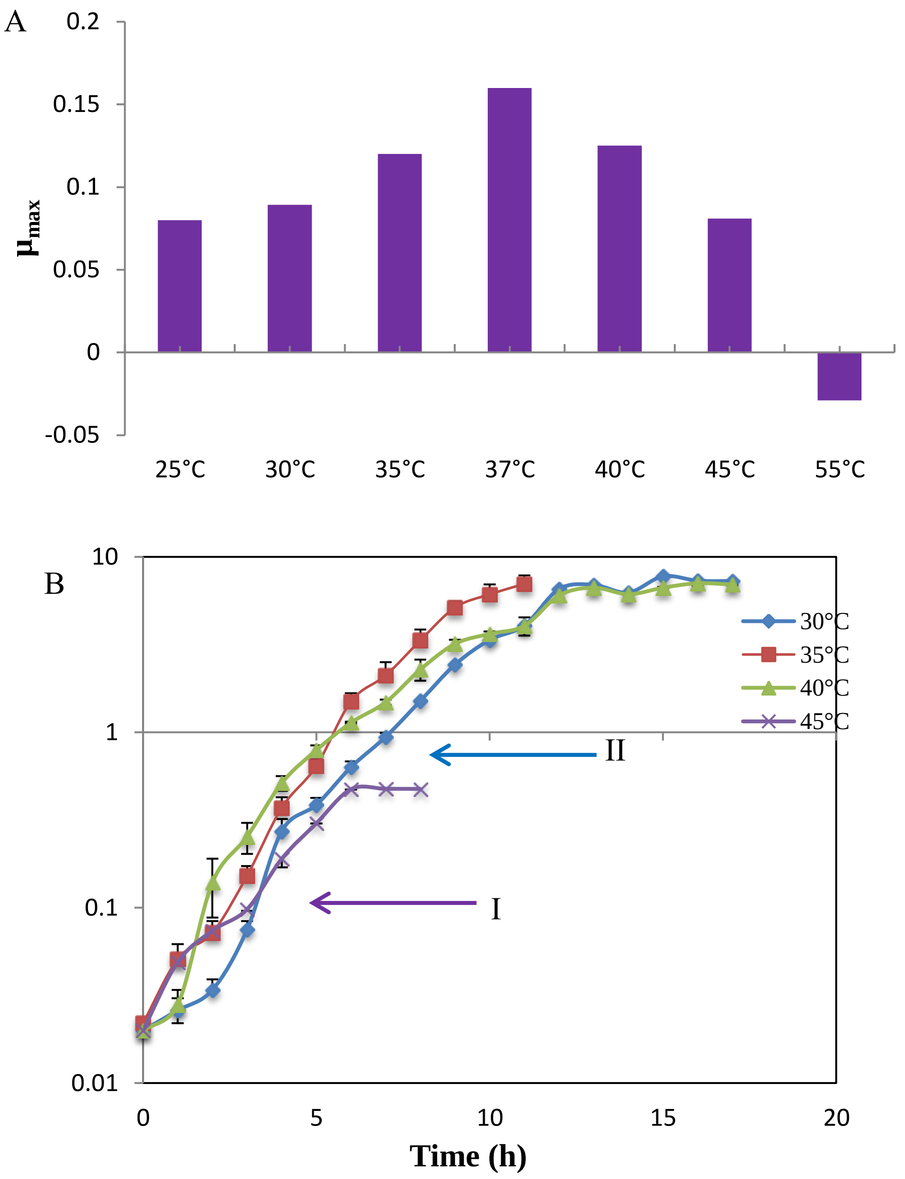

S1 Fig. (A) **Maximum specific growth rates of *Lb. casei* GCRL163 in MRS broth at different temperatures**. (B) **Growth curves of *Lb. casei* GCRL163 at 30°C, 35°C, 40°C and 45°C. Arrow I and II represent the points of harvest at mid-exponential phase for 45°C and other temperatures (30°C, 35°C and 40°C) respectively**. Bacterial cells for proteomic studies were harvested at mid-log [7 h (30°C), 6 h (35°C), 5 h (40°C) and 4 h (45°C)] corresponding to OD_{600} 1.04, 1.50, 0.69 and 0.19 respectively while cell samples for fatty acid compositional profiles were harvested at mid-exponential, late-exponential and stationary phases. Preliminary experiments indicated growth at 37°C as optimal temperature and no significant growth was observed beyond 50°C.
