# Supplementary material for: Proteomic analysis of Lactobacillus casei GCRL163 cell-free extracts reveals a SecB homolog and other biomarkers of prolonged heat stress
Source: PLoS One. 2018 Oct 25;13(10):e0206317. doi: 10.1371/journal.pone.0206317 (PMC6201924; doi:10.1371/journal.pone.0206317)
Supplement: S2 Fig — (PDF) [file pone.0206317.s006.pdf]

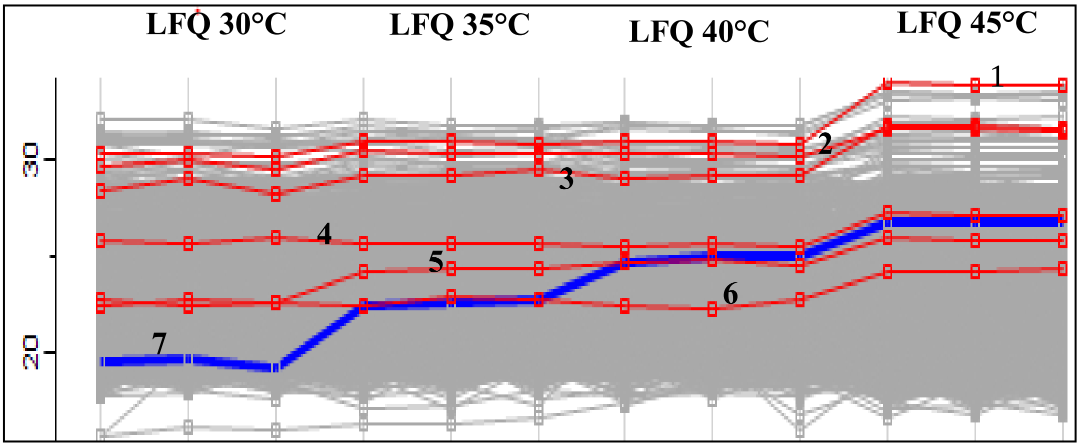

S2 Fig. **Abundance of selected chaperone proteins in triplicate samples of CFEs for cells harvested at mid-exponential growth at temperatures from 30 to 45°C.** LFQ data is log_2 transformed (Y-axis). 1, GroEL; 2, GroES; 3, DnaK; 4, ClpB; 5, DnaJ; 6, hsp33 and 7, BN194_29440. Note that BN194_29440 (which contains an α-crystallin domain of the Hsp20 family chaperones) is more highly abundant as the temperature increases, whereas the other molecular chaperones are elevated primarily at 45°C.
