# Supplementary material for: Proteomic analysis of Lactobacillus casei GCRL163 cell-free extracts reveals a SecB homolog and other biomarkers of prolonged heat stress
Source: PLoS One. 2018 Oct 25;13(10):e0206317. doi: 10.1371/journal.pone.0206317 (PMC6201924; doi:10.1371/journal.pone.0206317)
Supplement: S3 Fig — (PDF) [file pone.0206317.s007.pdf]

S3 Fig. Sequence alignment for selected SecB proteins using Clustal Omega (multiple-sequence) and Needle (pair-wise comparisons) (EMBL-EBI, www.ebi.ac.uk). Cladogram and phylogenetic tree were also constructed (EMBL-EBI tools suite and www.phylogeny.fr/).

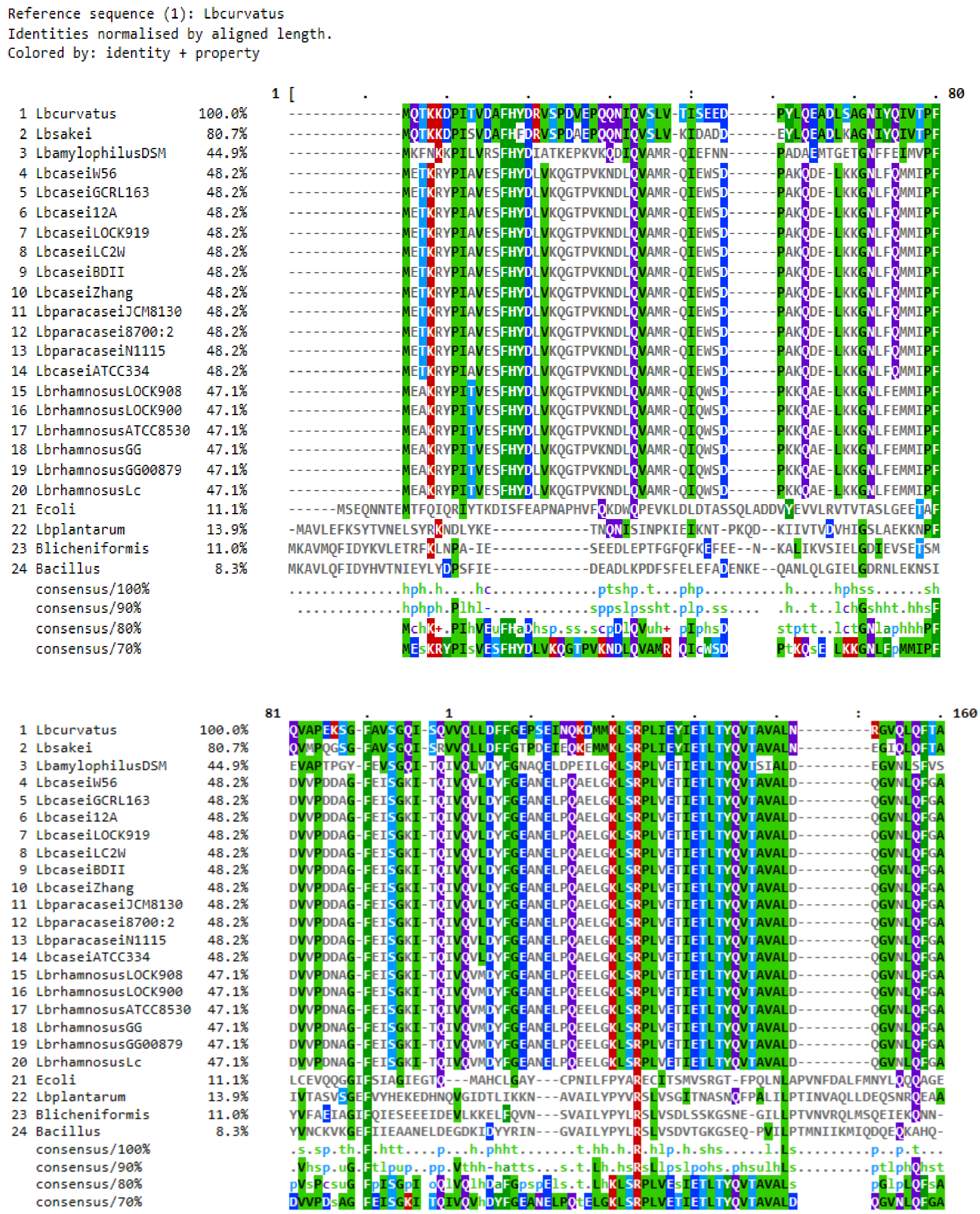

(A) Multiple sequence alignment. FASTA sequences were obtained by identifying nearest neighbours (K number bits down to 133, all *Lactobacillus* species) by KEGG BLAST of the GCRL163 SecB homolog (protein sequence from the RAST file of the sequenced genome). Other proteins were identified in

UniProtKG searches for ‘SecB’ then by genus (*Escherichia*, *Bacillus*, *Lactobacillus*), selecting a reviewed entry representative of Gram negative SecB (*E. coli* K12 protein ID P0AG86, where all top 50 hits for *E. coli* were 100% identical to this protein), two proteins from Gram positive species with submitted but unreviewed name of ‘Preprotein translocase subunit SecB’ (*Bacillus* sp. CC120222-01 protein A0A1X7ETD2, *Lb. plantarum* subsp. *plantarum* ATCC14917 protein D7V892), and an uncharacterised *Bacillus* protein with a domain structure similar to the *Lb. casei* domains from (*Bacillus licheniformis CG-B52* uncharacterised protein T5HC28, with overlapping homologous domains DUF1149, SecB [IPR003708] and SecB-like superfamily [IPR035958]) for comparison.

Where full names are not seen and not defined above, they correspond to: 3 = *Lb. amylophilus* DSM 20533; 18 = *Lb. rhamnosus* GG gene locus LRHM_0836 NCBI protein ID BAI41363; 19 = *Lb. rhamnosus* GG gene locus LGG_00879 NCBI protein ID CAR86774 (DUF1149 domain); 20 = *Lb. rhamnosus* Lc 705; 24 = *Bacillus* sp. CC120222-01.

```
                                    161        ] 169
 1 Lbcurvatus            100.0%         QANED----
 2 Lbsakei                80.7%         HETPEEA--
 3 LbamylophilusDSM       44.9%         GESEQAGE-
 4 LbcaseiW56             48.2%         SDEQPGQTK
 5 LbcaseiGCRL163         48.2%         SDEQPGQTK
 6 Lbcasei12A             48.2%         SDEQPGQTK
 7 LbcaseiLOCK919         48.2%         SDEQPGQTK
 8 LbcaseiLC2W            48.2%         SDEQPGQTK
 9 LbcaseiBDII            48.2%         SDEQPGQTK
10 LbcaseiZhang           48.2%         SDEQPGQTK
11 LbparacaseiJCM8130     48.2%         SDEQPGQTK
12 Lbparacasei8700:2      48.2%         SDEQPGQTK
13 LbparacaseiN1115       48.2%         SDEQPGQTK
14 LbcaseiATCC334         48.2%         SDEQPGQTK
15 LbrhamnosusLOCK908     47.1%         SDEQPGQA-
16 LbrhamnosusLOCK900     47.1%         SDEQPGQA-
17 LbrhamnosusATCC8530    47.1%         SDEQPGQA-
18 LbrhamnosusGG          47.1%         SDEQPGQA-
19 LbrhamnosusGG00879     47.1%         SDEQPGQA-
20 LbrhamnosusLc          47.1%         SDEQPGPA-
21 Ecoli                  11.1%         GTEEHQDA-
22 Lbplantarum            13.9%         D--------
23 Blicheniformis         11.0%         ---------
24 Bacillus                8.3%         ---------
   consensus/100%                       .........
   consensus/90%                        t........
   consensus/80%                        sppp.t...
   consensus/70%                        SDEQPGps.
```

(B) Phylogram and phylogenetic tree (hierarchical clustering, http://www.phylogeny.fr/)

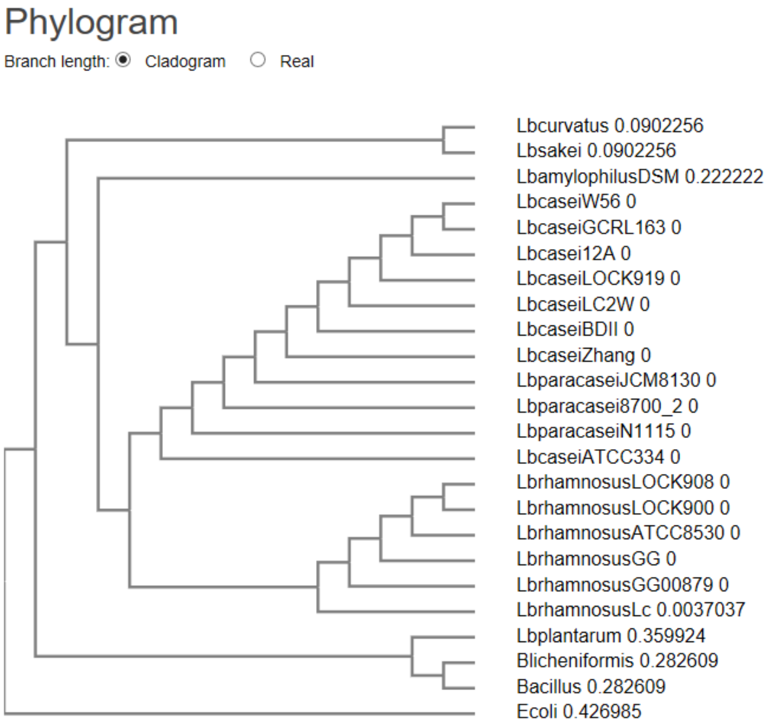

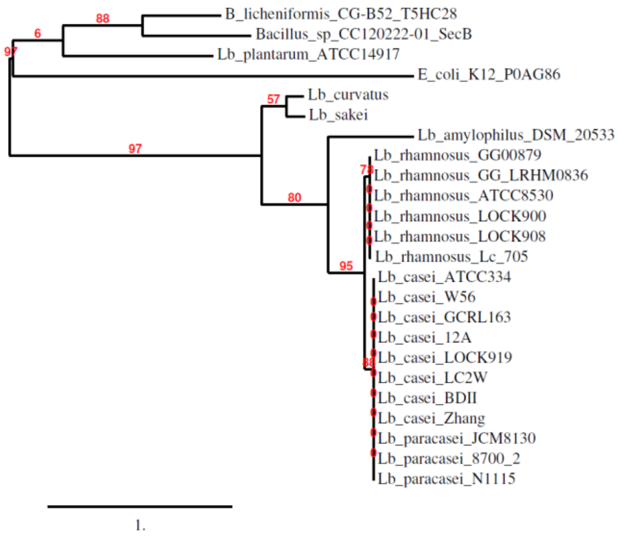

(C) Pair-wise sequence alignment by Needle in the EMBL-EBI toolkit (www.ebi.ac.uk), default settings.

(i) *E. coli* K12 P0AG86 SecB protein with *Lb. casei* GCRL163 uncharacterised protein (equivalent to W56 protein K0N3D6, BN194_10000) with DUF1149 and SecB-like superfamily domain (IPR035958), sequence from RAST file of the genome sequence.

```
########################################
# Program: needle
# Rundate: Tue 20 Mar 2018 01:37:53
# Commandline: needle
#    -auto
#    -stdout
#    -asequence emboss_needle-I20180320-013751-0686-70871265-p1m.asequence
#    -bsequence emboss_needle-I20180320-013751-0686-70871265-p1m.bsequence
#    -datafile EBLOSUM62
#    -gapopen 10.0
#    -gapextend 0.5
#    -endopen 10.0
#    -endextend 0.5
#    -aformat3 pair
#    -sprotein1
#    -sprotein2
# Align_format: pair
# Report_file: stdout
########################################

#=======================================
#
# Aligned_sequences: 2
# 1: E.
# 2: Lb.
# Matrix: EBLOSUM62
# Gap_penalty: 10.0
# Extend_penalty: 0.5
#
# Length: 217
# Identity:      17/217 ( 7.8%)
# Similarity:    30/217 (13.8%)
# Gaps:         143/217 (65.9%)
# Score: 16.0
#
#
#=======================================

E.                 1 --------------------------------------------------      0
                                                                        
Lb.                1 METKRYPIAVESFHYDLVKQGTPVKNDLQVAMRQIEWSDPAKQDELKKGN     50

E.                 1 ----------MSEQNNTEMTFQI-QRIYTKDISFEAPNAPHVFQKDWQPE     39
                               :.:....|::.:| |.:...|...||...|          
Lb.               51 LFQMMIPFDVVPDDAGFEISGKITQIVQVLDYFGEANELP----------     90

E.                40 VKLDLDTASSQLADDVYEVVLRVTVTA-SLGEETAFLCEVQQGGIFSIAG     88
                      :.:|...|..|.:.:..:..:||..| ..|....|....:|.|...   
Lb.               91 -QAELGKLSRPLVETIETLTYQVTAVALDQGVNLQFGASDEQPGQTK---    136

E.                89 IEGTQMAHCLGAYCPNILFPYARECITSMVSRGTFPQLNLAPVNFDALFM    138
                                                                        
Lb.              137 --------------------------------------------------    136

E.               139 NYLQQQAGEGTEEHQDA    155
                                      
Lb.              137 -----------------    136


#---------------------------------------
#---------------------------------------
```

(ii) *E. coli* K12 P0AG86 SecB protein with *Bacillus* sp. CC120222-01 protein A0A1X7ETD2, with submitted but unreviewed name of 'Preprotein translocase subunit SecB'.

```
########################################
# Program: needle
# Rundate: Tue 20 Mar 2018 01:40:54
# Commandline: needle
#    -auto
#    -stdout
#    -asequence emboss_needle-I20180320-014053-0238-62605872-p1m.asequence
#    -bsequence emboss_needle-I20180320-014053-0238-62605872-p1m.bsequence
#    -datafile EBLOSUM62
#    -gapopen 10.0
#    -gapextend 0.5
#    -endopen 10.0
#    -endextend 0.5
#    -aformat3 pair
#    -sprotein1
#    -sprotein2
# Align_format: pair
# Report_file: stdout
########################################

#=======================================
#
# Aligned_sequences: 2
# 1: EcoliK12SecB
# 2: BacillusspCC120222-01SecB
# Matrix: EBLOSUM62
# Gap_penalty: 10.0
# Extend_penalty: 0.5
#
# Length: 169
# Identity:      28/169 (16.6%)
# Similarity:    57/169 (33.7%)
# Gaps:          42/169 (24.9%)
# Score: 48.0
#
#
#=======================================

EcoliK12SecB       1 MSEQNNTEMTFQIQRIYTKDISFEAPNAPHVF-------QKDWQPEVKLD     43
                                  .:...:.|.:...|..:::       :.|.:|:...:
BacillusspCC1      1 -------------MKAVLQFIDYHVTNIEYLYDPSFIEDEADLKPDFSFE     37

EcoliK12SecB      44 LDTASSQLADDVYEVVLRVTVTASLG----EETAFLCEVQQGGIFSIAGI     89
                     |     :.||:..|.. .:.:...||    |:.:.....:..|.|.|...
BacillusspCC1     38 L-----EFADENKEQA-NLQLGIELGDRNLEKNSIYVNCKVKGEFIIEAA     81

EcoliK12SecB      90 EGTQMAHCLGAYCPN---ILFPYARECITSMVSRGTFPQLNLAPVNFDAL    136
                     ........:..|..|   ||:||.|..::.:..:|: .|..:.|.
BacillusspCC1     82 NELDEGDKIDYYRINGVAILYPYLRSLVSDVTGKGS-EQPVILPT-----    125

EcoliK12SecB     137 FMNYLQQQAGEGTEEHQDA    155
                      ||.::....:..:.||
BacillusspCC1    126 -MNIIKMIQDQEQKAHQ--    141
```

(iii) *E. coli* K12 P0AG86 SecB protein with *Lb. plantarum* subsp. *plantarum* ATCC14917 protein D7V892, with submitted but unreviewed name of 'Preprotein translocase subunit SecB'.

```
########################################
# Program: needle
# Rundate: Tue 20 Mar 2018 01:44:03
# Commandline: needle
#    -auto
#    -stdout
#    -asequence emboss_needle-I20180320-014401-0184-40768158-p1m.asequence
#    -bsequence emboss_needle-I20180320-014401-0184-40768158-p1m.bsequence
#    -datafile EBLOSUM62
#    -gapopen 10.0
#    -gapextend 0.5
#    -endopen 10.0
#    -endextend 0.5
#    -aformat3 pair
#    -sprotein1
#    -sprotein2
# Align_format: pair
# Report_file: stdout
########################################

#=======================================
#
# Aligned_sequences: 2
# 1: EcoliK12SecB
# 2: LbplantarumATCC14917
# Matrix: EBLOSUM62
# Gap_penalty: 10.0
# Extend_penalty: 0.5
#
# Length: 170
# Identity:      36/170 (21.2%)
# Similarity:    65/170 (38.2%)
# Gaps:          43/170 (25.3%)
# Score: 51.5
#
#
#=======================================

EcoliK12SecB       1 -----MSEQNNTEMTFQIQRIY---TKDIS----FEAPNAPHVFQKDWQP     38
                          .......|::::...:|   .::||    .|..|.|.      |.
LbplantarumAT      1 MAVLEFKSYTVNELSYRKNDLYKETNQNISINPKIEIKNTPK------QD     44

EcoliK12SecB      39 EVKLDLDTASSQLADDVYEVVLRVTVTASLGEETAF--LCEVQQGGIFSI     86
                     ::.:.:|.....||:.....:    ||||:..|..:  ..:..|.||.::
LbplantarumAT     45 KIIVTVDVHIGSLAEKKNPFI----VTASVSGEFVYHEKEDHNQVGIDTL     90

EcoliK12SecB      87 AGIEGTQMAHCLGAYCPNILFPYARECITSMVSRGT-FPQLNLAPVNFDA    135
                       |:...:|         ||:||.|..::.:.:... ||.|.|..:|...
LbplantarumAT     91 --IKKNAVA---------ILYPYVRSLVSGITNASNQFPALILPTINVAQ    129

EcoliK12SecB     136 LFMNYLQQQAGEGTEEHQDA    155
                     |    |.:|:..  :|..| 
LbplantarumAT    130 L----LDEQSNR--QEAAD-    142
```

(iv) *E. coli* K12 P0AG86 SecB protein with *Bacillus licheniformis CG-B52* uncharacterised protein T5HC28, with overlapping homologous domains DUF1149, SecB (IPR003708) and SecB-like superfamily (IPR035958).

```
########################################
# Program: needle
# Rundate: Tue 20 Mar 2018 05:25:35
# Commandline: needle
#    -auto
#    -stdout
#    -asequence emboss_needle-I20180320-052534-0238-65757154-p1m.asequence
#    -bsequence emboss_needle-I20180320-052534-0238-65757154-p1m.bsequence
#    -datafile EBLOSUM62
#    -gapopen 10.0
#    -gapextend 0.5
#    -endopen 10.0
#    -endextend 0.5
#    -aformat3 pair
#    -sprotein1
#    -sprotein2
# Align_format: pair
# Report_file: stdout
########################################

#=======================================
#
# Aligned_sequences: 2
# 1: EcoliK12P0AG86
# 2: BlicheniformisCG-B52
# Matrix: EBLOSUM62
# Gap_penalty: 10.0
# Extend_penalty: 0.5
#
# Length: 184
# Identity:      28/184 (15.2%)
# Similarity:    50/184 (27.2%)
# Gaps:          75/184 (40.8%)
# Score: 54.0
#
#
#=======================================

EcoliK12P0AG8      1 ----------------------MSEQNNTEMTFQIQRIYTKDISFEAPNA     28
                                           :..:.:.|.||..|              
Blicheniformi      1 MKAVMQFIDYKVLETRFKLNPAIESEEDLEPTFGFQ--------------     36

EcoliK12P0AG8     29 PHVFQKDWQPEVKLDLDTASSQLADDVYEVVLRVTVTASLGE----ETAF     74
                         .|::....|                   :.|:..||:    ||:.
Blicheniformi     37 ----FKEFEENKAL-------------------IKVSIELGDIEVSETSM     63

EcoliK12P0AG8     75 LCEVQQGGIFSIAG---IEGTQMAHCLGAYCPNILFPYARECITSMVSRG    121
                     ....:..|||.|..   |:..............||:||.|..::.:.|:|
Blicheniformi     64 YVFAEIAGIFQIESEEEIDEVLKKELFQVNSVAILYPYLRSLVSDLSSKG    113

EcoliK12P0AG8    122 TFPQLNLAPVNFDALFMNYLQQQAGEGTEEHQDA    155
                     :...:.|..||...|....:::|..         
Blicheniformi    114 SNEGILLPTVNVRQLMSQEIEKQNN---------    138


#---------------------------------------
```
